# Antagonistic roles by the conserved nuclear poly(A)-binding proteins PABPN1 and ZC3H14 in nuclear RNA surveillance

**DOI:** 10.1093/nar/gkaf060

**Published:** 2025-02-03

**Authors:** Mélodie Latour, Lauren Kwiatek, Anne-Marie Landry-Voyer, François Bachand

**Affiliations:** RNA Group, Department of Biochemistry & Functional Genomics, Université de Sherbrooke, Sherbrooke, Québec, J1E 4K8, Canada; RNA Group, Department of Biochemistry & Functional Genomics, Université de Sherbrooke, Sherbrooke, Québec, J1E 4K8, Canada; RNA Group, Department of Biochemistry & Functional Genomics, Université de Sherbrooke, Sherbrooke, Québec, J1E 4K8, Canada; RNA Group, Department of Biochemistry & Functional Genomics, Université de Sherbrooke, Sherbrooke, Québec, J1E 4K8, Canada

## Abstract

Most eukaryotic genomes are transcribed pervasively, thereby producing an array of long non-coding RNAs (lncRNAs) in addition to protein-coding mRNAs. A large fraction of these lncRNAs is targeted by polyadenylation-dependent decay via the poly(A)-binding protein nuclear 1 (PABPN1) and the RNA exosome. Yet, how PABPN1 contributes to nuclear RNA surveillance by facilitating lncRNA turnover by the RNA exosome remains largely unclear. Here, we show that PABPN1 is important for the nuclear retention of polyadenylated lncRNAs, such that PABPN1 loss of function allows target lncRNAs to evade nuclear decay, leading to cytoplasmic accumulation. Interestingly, we found that another nuclear PABP, ZC3H14, functions antagonistically to PABPN1 and the poly(A)-tail exosome targeting (PAXT) connection in the control of nuclear lncRNA turnover. Collectively, our findings disclose the critical interplay between two conserved nuclear PABPs, PABPN1 and ZC3H14, in RNA surveillance via the control of nuclear RNA export.

## Graphical Abstract

**Graphical Abstract:**
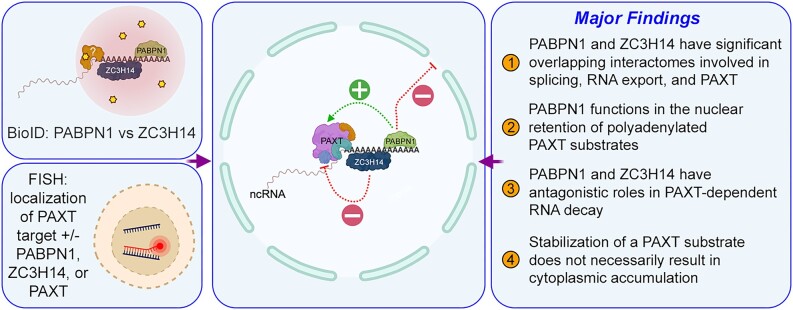

## Introduction

The addition of a polyadenylate stretch (poly(A) tail) at the 3′ end of nascent RNAs is a fundamental step of gene expression for most protein-coding and many non-coding (nc) transcripts. The 3′ end poly(A) tail is important for nuclear export, mRNA stability, and translation control [1]. RNA polyadenylation is a two-step process that includes (i) endonucleolytic cleavage of the nascent transcript and (ii) adenosine polymerization onto the free 3′ hydroxyl group of the cleaved RNA [2]. Cleavage of the nascent transcript is normally completed co-transcriptionally by the 3′ end processing machinery, a multi-subunit complex that includes an evolutionarily conserved endonuclease. 3′ end polyadenylation is then catalyzed by a conserved poly(A) polymerase (PAP) module that is flexibly associated to the 3′ end processing machinery complex [3]. Although recombinant human PAP is sufficient to catalyze 3′ end polyadenylation *in vitro*, the addition of the poly(A)-binding protein nuclear 1 (PABPN1) strongly stimulates PAP processivity and poly(A) tail length [4, 5]. Stimulation of PAP activity is mediated by a helical domain in the N-terminal half of PABPN1 [6], while polyadenosine binding is mediated via a central RNA recognition motif and the C-terminal arginine-rich region [7]. Consistent with these biochemical studies, poly(A) tail length analysis of newly synthesized RNAs from PABPN1-depleted human cells reveals generally shorter poly(A) tails, supporting a role for PABPN1 in stimulating 3′ end polyadenylation *in vivo* [8].

In addition to its stimulatory role in the 3′ end polyadenylation of nascent transcripts, studies over the past decade have uncovered an unexpected function for PABPN1 in polyadenylation-dependent RNA decay. The first results linking PABPN1 to nuclear RNA decay were reported in the fission yeast, *Schizosaccharomyces pombe*. In this organism, the PABPN1 homolog, Pab2, was shown to contribute to a polyadenylation-dependent mechanism of RNA decay that was mediated by the RNA exosome complex of 3′-5′ exonucleases, which targeted various gene biotypes, including small nucleolar RNAs [9], early meiotic genes [10], inefficiently spliced genes [11], and retrotransposons [12]. Subsequently, transcriptome analysis of human cells by RNA-seq identified a class of PABPN1-sensitive ncRNAs, the large majority of which accumulated in conditions of PABPN1 deficiency [13]. This included many spliced long non-coding RNAs (lncRNAs) expressed from snoRNA host genes (SNHG) as well as the well-studied NEAT1 lncRNA [13]. The mechanism by which PABPN1 promotes exosome-mediated RNA decay in the nucleus involves PAP-dependent RNA hyperadenylation [14] and functional associations with nuclear proteins, including MTR4, ZFC3H1, ZC3H3, and RBM26/27 [15–17]. This nuclear exosome adaptor function is known as the poly(A)-tail exosome targeting (PAXT) connection [15]. Consistent with the evolutionarily conserved role of *S. pombe* Pab2 and human PABPN1 in polyadenylation-dependent nuclear RNA decay, the PAXT connection has a *S. pombe* counterpart known as MTREC (Mtl1-Red1 core) that involves homologs of human MTR4 (Mtl1), ZFC3H1 (Red1), ZC3H3 (Red5), and RBM26/27 (Rmn1) [18–20]. How Pab2/PABPN1 contribute to MTREC/PAXT-dependent nuclear RNA degradation remains unknown, however.

Although PABPN1 is the primary nuclear PABP in most eukaryotic organisms, the discovery and characterization of novel RNA-binding proteins has expanded the current list of PABP family members [21]. The Zinc finger Cys/Cys/Cys/His protein 14 (ZC3H14) is one evolutionarily conserved PABP that has been identified in *S. cerevisiae* [22], *S. pombe* [23], *Drosophila* [24], and humans [25]. In addition to the presence of CCCH zinc finger domains that are responsible for binding to polyadenosine RNA [26], ZC3H14 and its homologs all share an N-terminal proline-tryptophan-isoleucine-like fold, a central glutamine-rich region, and nuclear localization signals [27]. The *S. cerevisiae* homolog of human ZC3H14, Nab2, has been the most extensively studied CCCH zinc finger PABP to date. *S. cerevisiae NAB2* encodes an essential protein [22] that was shown to be important for mRNA export and poly(A) tail length control [28, 29]. Rapid nuclear depletion of Nab2 results in transcriptome-wide decay of nascent polyadenylated RNAs by the RNA exosome [30], indicating that binding of Nab2 to nascent poly(A) tails protects RNAs from nuclear degradation. Consistent with the role of Nab2 in protecting nascent RNAs from nuclear decay, blocking nuclear export induces the degradation of newly synthesized polyadenylated transcripts, an outcome that can be rescued by the overexpression of Nab2 [31]. In addition to protecting newly-synthesized transcripts from RNA decay, binding of Nab2 to nascent poly(A) tails also controls poly(A) tail length by inhibiting the poly(A) polymerase Pap1 [2]. Similar to a Nab2 loss of function in budding yeast, knockdown of ZC3H14 in a human neuronal cell line results in RNAs with longer poly(A) tails [32]. In cultured human cells, ZC3H14 primarily localizes to nuclear speckles [25] and associates with spliceosome components [33]. Interestingly, a recent study revealed that ZC3H14 phosphorylation regulates its association with PAXT and promotes the nuclear degradation of transcripts that are prematurely terminated during RNA polymerase II transcription [34].

To date, little is known about the potential regulatory interplay existing between nuclear PAPBs. In fission yeast, Nab2 was shown to prevent Pab2-dependent exosome-mediated decay of an inefficiently spliced polyadenylated pre-mRNA [23]. However, whether similar functional interactions exist between human ZC3H14 and PABPN1 remain unknown. In this study, we found that PABPN1 and ZC3H14 copurify and associate with a large set of common RNA-binding proteins. We also show that ZC3H14 antagonistically regulates a set of non-coding transcripts that are targeted for nuclear RNA decay via PAXT and PABPN1. Notably, our data revealed that PABPN1 functions in the nuclear retention of PAXT substrates, leading to marked cytoplasmic lncRNA accumulation in PABPN1-deficient cells. When ZC3H14 was depleted together with PABPN1, the cytoplasmic accumulation of a PAXT substrate resulting from a PABPN1 loss of function was significantly reduced. Our findings therefore disclose antagonistic roles for the conserved nuclear PABPs, ZC3H14 and PABPN1, in PAXT-dependent nuclear surveillance.

## Materials and methods

### Cell culture

Cell cultures were maintained in an incubator at a temperature of 37°C with 5% CO_2_ and high humidity. HeLa, HEK293T, HEK 293-FT, HeLa-shNT, and HeLa-shPABPN1 cells were cultured in Dulbecco's Modified Eagle's Medium (DMEM) supplemented with 10% fetal bovine serum (FBS) and, respectively, 1% penicillin-streptomycin or 10 μg/ml zeocin and 100 μg/ml blasticidin or 2ug/mL of puromycin. Inducible expression of GFP-ZC3H14, BirA-ZC3H14, GFP-PABPN1, and BirA-PABPN1 was achieved by flippase-mediated recombination in HEK 293-FT cells as previously described [35]. Briefly, PABPN1 cDNA was amplified by polymerase chain reaction (PCR) using forward and reverse primers containing attB sequences. The resulting product was introduced into the pDONR221 vector using BP clonase II-mediated recombination (Life Technologies). The cDNA was then inserted into the pgLAP1-GFP / BirA plasmid using LR Clonase II (Life Technologies). ZC3H14 cDNA was amplified by PCR using forward and reverse primers containing homology sequences with the pgLAP1-GFP / BirA or pcDNA 5/FTR/TO plasmid prior to the Gibson assembly (NEB). The resulting constructs were co-transfected into HEK 293-FT cells with pOG44 expressing Flp recombinase. Positively integrated cells were selected with 150 μg/ml hygromycin and 15 μg/ml basticidin. Positive clones were maintained in DMEM supplemented with 10% FBS, 75 μg/ml hygromycin, and 15 μg/ml blasticidin. Expression of the tagged proteins and shRNAs was obtained with 500 ng/ml of doxycycline for 48–72 h. siRNAs (Supplementary Table S1) were transfected with Lipofectamine 2000 (Thermo Fisher) at a final concentration of 25 nm for 72 h. Although we acknowledge that siRNA-mediated depletions have the potential to result in secondary effects, all siRNAs used in the current study have been designed to minimize off-target effects. Accordingly, we selected sequences with low homology to other genes, occasionally used pooled siRNAs to dilute potential off-targets effects of any single siRNA, and always included a non-targeting control siRNA to distinguish specific from non-specific effects.

### Proximity-dependent biotinylation (PDB-MS)

HEK 293-FT cells expressing BirA-tagged versions of proteins were seeded in a 15-cm dish. Induction of BirA-tagged proteins was obtained with 50 μm biotin for 24 h. Cells were collected (confluence >90%) in lysis buffer (50 mM Tris-HCl [pH 7.5], 150 mM NaCl, 1.5 mM MgCl2, 1mM EGTA, 0.1% SDS, 1% IGEPAL supplemented with 1 mM dithiothreitol [DTT], 1X protease inhibitor cocktail (Roche), 1 mM phenylmethylsulfonyl fluoride (PMSF), and 0.4% sodium deoxycholate) and incubated at 4°C for 20 min. The lysates were subsequently sonicated three times for 10 s and incubated at 4°C for 15 min with 167 units/ml of Benzonase (Sigma, E1014). The mixture was centrifuged for 20 min at 5000 rpm at 4°C. An equal amount of proteins was incubated with streptavidin sepharose beads (Sigma) for 3 h at 4°C. The samples were then centrifuged for 1 min at 2000 rpm. The beads were washed once with washing buffer (50 mM Tris HCl [pH 7.5], 2% SDS) then three times with lysis buffer supplemented with DTT. The samples were subsequently washed five times with 50 mM ammonium bicarbonate (ABC) buffer [pH 8.5]. An on-bead protease digestion was performed. Briefly, 10 mM DTT in 20 mM ABC was added to the beads and incubated for 30 min at 60°C, followed by 15 mM iodooacetamide (IAA) in 20 mM ABC and the mixture was incubated for 1 h in the dark. Around 50 ng of trypsin was added to the beads at 37°C for an overnight digestion. Tryptic digestion was then stopped using 1% formic acid (FA). The eluates were then concentrated using a speedvac and resuspended in a buffer consisting of 0.1% trifluoroacetic acid. The samples were then purified using ZipTip and taken up in 1% FA for LC–MS/MS.

### LC-MS/MS analysis

Trypsin-digested peptides were separated using a Dionex Ultimate 3000 nanoHPLC system. Ten microliters of sample (a total of 2 μg) in 1% (v/v) FA were loaded with a constant flow of 4 μl/min onto an Acclaim PepMap100 C18 column (0.3 mm id × 5 mm, Dionex Corporation). After trap enrichment, peptides were eluted onto an EasySpray PepMap C18 nano column (75 μm × 50 cm, Dionex Corporation) with a linear gradient of 5–35% solvent B (90% acetonitrile with 0.1% FA) over 240 min with a constant flow of 200 nl/min. The HPLC system was coupled to an OrbiTrap QExactive mass spectrometer (Thermo Fisher Scientific Inc) via an EasySpray source. The spray voltage was set to 2.0 kV and the temperature of the column set to 40°C. Full scan MS survey spectra (*m*/*z* 350–1600) in profile mode were acquired in the Orbitrap with a resolution of 70 000 after accumulation of 1 000 000 ions. The ten most intense peptide ions from the preview scan in the Orbitrap were fragmented by collision-induced dissociation (normalized collision energy 35% and resolution of 17 500) after the accumulation of 50 000 ions. Maximal filling times were 250 ms for the full scans and 60 ms for the MS/MS scans. Precursor ion charge state screening was enabled and all unassigned charge states as well as singly, 7 and 8 charged species were rejected. The dynamic exclusion list was restricted to a maximum of 500 entries with a maximum retention period of 40 s and a relative mass window of 10 ppm. The lock mass option was enabled for survey scans to improve mass accuracy. Data were acquired using the Xcalibur software version 4.1. Peptide identification was performed with MaxQuant version 1.6.2.2 software using the human proteome from Uniprot.

### Protein analyses and antibodies

Cells were washed with phosphate-buffered saline (PBS) and then collected in 1.5-ml tubes. Seventy percent of the cells was kept for RNA extraction, and 30% was used for protein extraction. For all experiments, the cell pellet was resuspended in lysis buffer (50 mM Tris-HCl, pH 7.5, 150 mM NaCl, 0.1% Triton X-100, 10% glycerol, 2 mM MgCl2, 1 mM dithiothreitol [DTT], and 1X complete protease inhibitor cocktail [Roche]) following harvest and incubated at 4°C for 20 min. The mixture was centrifuged for 15 min at 13 000 rpm. The supernatant was transferred into a new tube, loading dye was added to a final 1X concentration (62.5 mM Tris-HCl, pH 6.8, 10% glycerol, 2% SDS [vol/vol], 0.1 M DTT, and bromophenol blue), and the mix was heated for 5 min at 95°C. Proteins were separated by Sodium dodecylsulphate-polyacrylamide gel electrophoresis (SDS-PAGE), transferred to nitrocellulose membranes, and analyzed by immunoblotting using the following primary antibodies: anti-PABPN1 (ab75855, 1:1000 (v/v); Abcam), anti-tubulin (T5168, 1:4000 (v/v); Sigma-Aldrich), anti-ZC3H14 (A303-993A, 1: 1000 (v/v); Bethyl Laboratories), anti-Flag (F3165, 1:1000 (v/v); Sigma-Aldrich), anti-RBM26 (A301-216A, 1:1000 (v/v); Bethyl Laboratories), anti-PCF11 (A303-706A, 1:1000 (v/v); Bethyl Laboratories); anti-UAP56 (A8356, 1:1000 (v/v); ABclonal) and anti-GFP (11 814 460 001, 1:1000 (v/v); Roche). The membranes were then incubated with IRDye 800CW-conjugated donkey anti-rabbit antibody (926–32 213, 1:15 000 (v/v); LI-COR) and AlexaFluor 680-conjugated goat anti-mouse antibody (A21057, 1:15 000; Life Technologies). Protein detection was performed using an Odyssey infrared imaging system (LI-COR). All quantitative protein analyses presented in this study were generated from at least three independent transfection/induction experiments, in which the data and error bars represent averages and standard deviations, respectively.

### RNA preparation and analysis

To assess the effect of ZC3H14 deficiency on *SNHG19* stability, HeLa cells previously transfected with control and ZC3H14–specific siRNAs were treated with 5 mg/ml actinomycin D, and RNA was isolated at time zero and intervals thereafter indicated. For all experiments, total RNA was extracted using TRIzol RNA isolation reagents (Thermo Fisher) and 1 μg of total RNA was treated with 1 unit of RNase-free DNase RQ1 (Promega, M6101) for 30 min at 37°C and inactivated with 1 μL of 25 mM EDTA for 10 min at 65°C. Reverse transcription reactions were in a volume of 20 μL using random hexamers and 2 units of RT (MMuLV Reverse Transcriptase) for 60 min at 42°C and inactivated for 20 min at 65°C. qPCR reactions were performed in triplicates on a LightCycler 96 system (Roche) in a final volume of 15 μL using 6 μL from a 1:20 dilution of each cDNA, 0.15 μM of forward and reverse primers, and 7.5 μL of the 2 × PerfeCTa SYBR Green SuperMix. Analysis of gene expression changes were calculated relative to the appropriate control samples and were measured with the ΔΔCT method using the *GAPDH* mRNA as an internal reference. The oligonucleotides used in the qPCR experiments are listed in Supplementary Table S2.

### Protein and RNA co-immunoprecipitation assays

HEK 293-FT cells expressing GFP, GFP-ZC3H14 or GFP-PABPN1 (confluence > 90%) were seeded in a 10-cm dish. Cells were washed with PBS and incubated 20 min in RNA co-immunoprecipitation (RIP) lysis buffer (50 mM Tris-HCl, pH 7.5, 150 mM NaCl, 1% Triton X-100, 2 mM MgCl2, 1 mM DTT, 10% glycerol, 40 U/ml RNase OUT [Life Technologies], and 1X complete protease inhibitor cocktail) with mixing at 4°C. Lysate was centrifuged at 13 000 RPM for 10 min at 4°C. To assess the requirement of RNA for protein–protein interactions, a fraction was treated for 15 min at room temperature with a mix of RNase A and T1 (62.5 μg/mL and 156 U/mL, respectively; ThermoFisher) or 3125 U/mL of RNase I (ThermoFisher). Part of the RNase-treated lysate was loaded onto a 0,8% agarose gel and revealed under UV light. For the RIP assays, 10% of the supernatant was kept for RNA extraction (input fraction) and 5% for protein analysis. The rest of the lysate then was incubated with 30 μl of GFP-TRAP beads for 3 h at 4°C. The beads were washed 3 times with RIP lysis buffer. Five percent of the immunoprecipitated RNA was kept for protein analysis. The remaining beads were subjected to RNA extraction using TRIzol reagent (Thermo Fisher) and reverse transcriptase-polymerase chain reaction (RT-qPCR) was performed using the SuperScript III reverse transcriptase (Invitrogen). qPCR reactions were performed as described previously.

### RNA–seq analysis

Libraries of control HeLa cells and those depleted for PABPN1 and/or ZC3H14 were prepared using total RNA and analyzed using Illumina HiSeq technology. We obtained 80 980 735, 80 045 534, 75 854 836, and 66 511 733 100-bp paired-end reads for control, PABPN1-, ZC3H14-, and PABPN1/ZC3H14-depleted cells, respectively. Paired-end fastq quality control, trimming and adapter-removal were performed with trim_galore v0.6.6 [36]. Reads were aligned against human genome version GRCh38 and annotated with gtf version 108 with Rsubread v2.10.5 [37]. Resulting ensembl gene annotation was matched with gene symbol, description, and EntrezID by using R package org.Hs.eg.db v3.16.0 [38]. Unsupervised MDS and heatmap plots were generated to assess the quality of sample libraries. Library size normalization (through TMM method) and lowly expressed gene filtering were performed using R package edgeR v3.40.2 [39].

### Immunofluorescence

HeLa cells were washed twice with PBS, fixed with 4% paraformaldehyde (PFA) for 15 min at room temperature, and washed twice again with PBS. Fixed cells were permeabilized using a 0.5% Triton X-100/PBS solution for 10 min and washed three times with PBS. Cells were blocked for 20 min in PBS with 1% BSA followed by a 1-h incubation with primary antibody dilution in PBS with 1% BSA (anti-PABPN1 (ab75855, 1:1000 (v/v); Abcam), anti-ZC3H14 (A303-993A, 1: 1000 (v/v); Bethyl Laboratories) and anti-SC35 (ab11826, 1:1000 (v/v); Abcam)). The cells were washed three times with PBS with 1% BSA and incubated with a secondary antibody dilution (mouse Alexa Fluor 568 1/500 (Invitrogen) and rabbit Alexa Fluor 488 1/500 (Abcam), in PBS with 1% BSA) for 1 h. Three washes were subsequently performed with PBS with 1% BSA before DAPI labeling (0.5 μg/mL) for 5 min followed by final washing with 1X PBS for 2 min. Slides were mounted on a coverslip with SlowFade Gold antifade solution (Life Technologies). Images were captured by Zeiss Axio Observer microscope using a 63 × oil objective.

### Fluorescence *in situ* hybridization (FISH) and immunofluorescence detection

The FISH signals were detected using Cy3-labeled probes. Briefly, HeLa cells were seeded on coverslips, and transfected with siRNAs for 72 h. Attached cells were washed twice with 1X PBS, fixed with 4% PFA for 20 min at room temperature, and washed twice again with 1X PBS. Cells were permeabilized in 70% ethanol at −20°C overnight and washed once with 2X SSC (0.3 M NaCl, 30 mM Trisodium citrate dihydrate, pH 7.0), and twice with 2X SSC, 10% formamide for 5 min at RT. For hybridization, each coverslip was incubated with 80 μl hybridization mix (10% formamide, 2X SSC, 0.5 mg/ml yeast tRNA, 10% dextran sulphate, 250 μg/ml BSA, 10 mM Ribonucleoside Vanadyl Complexes, 20 ng/μl Cya3 probes) at 37°C overnight in the dark. After hybridization, cells were washed twice with 2X SSC, 10% formamide for 15 min at 37°C. For FISH-coupled immunofluorescence detection, Cy3- hybridized cells were blocked in BBT (1X PBS, 0.3% Triton X-100, 0.5% BSA) for 1 h at RT. Each coverslip was incubated with 100μl of primary antibody (rabbit anti-Tubulin, 1:1000 in BBT, Sigma-Aldrich) at 4°C overnight, then washed three times with PBT (1X PBS, 0.3% Triton X-100) prior to incubation with 100 μl of secondary antibody (mouse Alexa Fluor 488, 1:1000 in BBT, Invitrogen). Before mounting, nuclei were stained with DAPI for 5 min at RT and mounted with SlowFade® Gold Antifade solution (Life Technologies). All images were captured with Confocal Zeiss LSM700 63X oil objective.

### RNAscope

RNA-FISH was performed with the RNAscope Multiplex Fluorescent V2 kit (Advanced Cell Diagnostics, Newark, CA) using HeLa cells. Briefly, cells were seeded on coverslips coated with poly-Lysine-D (Sigma #P6407) to obtain a final confluence of 50%. Cells were washed and fixed with 4% PFA for 30 min at room temperature, followed by dehydration with a gradient of 50/70/100% EtOH and rehydration of 70%/50% EtOH and 1 x PBS. Slides were then treated with hydrogen peroxide for 10 min at room temperature and washed twice with PBS (PBS + Tween20 0.1%). Slides were treated with protease III (diluted 1:15 in PBS) for 10 min at room temperature. Following three washes with PBS, slides were hybridized with RNAscope probes designed to detect *SNHG19* transcript (RNAscope HS-SNHG19-O1-C1). Hybridizations were performed at 40°C using homemade oven and labeled using the RNAscope Multiplex Fluorescent V2 protocol with TSA plus fluorophores (1:3000 dilution for Opal 520 (FP1487001KT; Akoya Bioscience)) as per the manufacturer's recommendations. Following the final wash, slides were counterstained with DAPI and mounted on coverslips using ProLong Gold antifade mounting media (ThermoFisher).

### Microscopy

Images were acquired using an LSM880 confocal microscope equipped with a Plan-Apochromat 40x/1.4 oil immersion objective with a 1.6x zoom (Zeiss) as described previously [40] for RNAscope and with Zeiss LSM700 for FISH and IF. Briefly, eight Z-sections of 1.54 μm were acquired for each condition and parameters acquisition times have been defined to avoid pixel saturation to ensure appropriate visualization and intensity quantification. These conditions have been adjusted in the same way between conditions. For display purposes, image sections have been deconvolved using the ZEN 2.6 iterative algorithm. A macro made with the software ImageJ [41] was used to cluster eight Z-sections to form a single image that is presented in the results section. Cropping and changes of 300-dpi resolution were made in Photoshop for data presentation. Image quantifications were performed using ImageJ, as previously described [40].

### Image quantification

Image quantifications were performed using ImageJ [41] and CellProfiler4.1.3 [42] software, as described previously [40]. Briefly, the processing of images from immunofluorescence experiments includes subtraction of the background at a cutoff of 30 and thresholding with the default filter. The SC35 signal was used to delineate the speckles on the nuclei showing the PABPN1 or ZC3H14 signal. A calculation of the intensity (mean grey value) was made using the ROI Manager. Processing of images from RNAscope experiments includes thresholding with Yen filter. The nucleus and cytoplasm were demarcated by DAPI, the latter by high exposure and subtraction of the former. A calculation of the intensity (mean grey value) was made using the ROI Manager. A nucleus/cytoplasm ratio was then measured based on signal intensity, and the measurements were exported to GraphPad Prism version 9 for statistical analyses.

## Results

### ZC3H14 and PABPN1 associate with a common set of proteins involved in RNA processing and polyadenylation-dependent RNA decay

**Figure 1.**
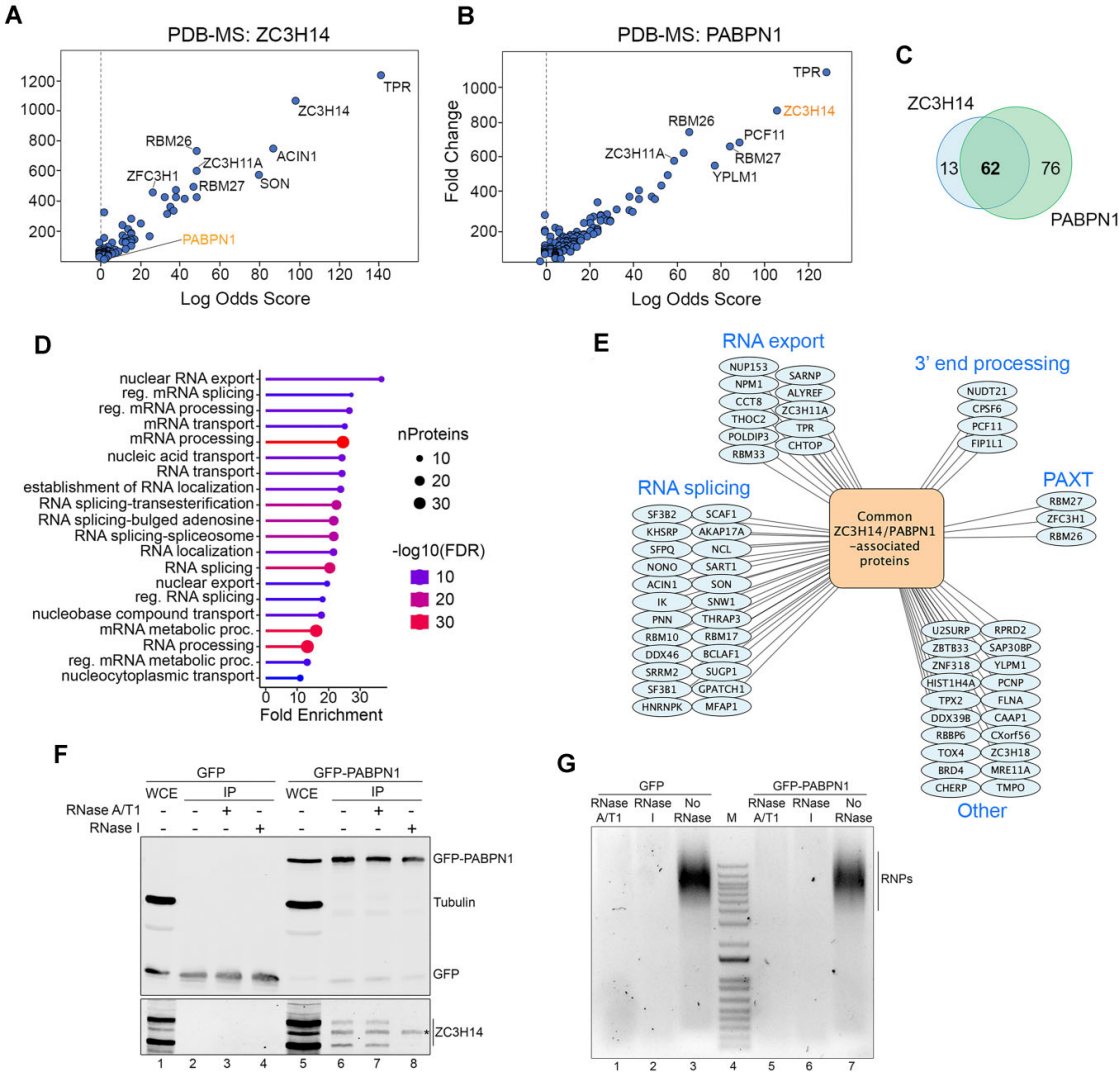
ZC3H14 and PABPN1 interact with a common set of RNA-binding proteins. (**A** and **B**) Scatterplots showing results from PDB-MS assays using BirA-ZC3H14 (A, *n* = 5) and BirA-PABPN1 (B, *n* = 3) plotted by Log Odds Score on the *x*-axis, which reflects the probability of a true protein–protein interaction; and fold change up the *y*-axis, reflecting relative enrichment in the streptavidin pull-down. Selected proteins are indicated. (**C**) Venn diagram of significant interactions partners detected by PDB-MS analysis of ZC3H14 and PABPN1 after running SAINTexpress. (**D**) GO enrichment analysis (biological process) of 62 overlapping proteins identified by PDB-MS analysis of ZC3H14 and PABPN1. The biological process and the number of proteins associated with each one are indicated. (**E**) Network of the common ZC3H14- and PABPN1-proximal interactome organized by biological processes found to be significantly enriched. (**F**) Western blot analysis of total extracts (lanes 1 and 5) and GFP immunoprecipitates (lanes 2–4 and 6–8) prepared from stable HEK293T cells expressing GFP (lanes 1–4) and GFP-PABPN1 (lanes 5–8). Total cell extracts were either treated (lanes 3–4 and 7–8) or not treated (lanes 2 and 6) with the indicated RNases before immunoprecipitation. Anti-GFP, anti-Tubulin, and anti-ZC3H14 were used for Western blotting. The asterisk indicates a non-specific protein that cross-reacts with the ZC3H14 antibody. (**G**) Agarose gel analysis of total extracts prepared from HEK293T cells expressing GFP (lanes 1–3) and GFP-PABPN1 (lanes 5–7) that were either treated (lanes 1–2 and 5–6) or not treated (lanes 3 and 7) with the indicated RNases to digest cellular RNAs. Cellular ribonucleoprotein (RNP) complexes are indicated on the right.

Our previous analysis of the human PABPN1 interactome by proximity-dependent biotinylation coupled to mass spectrometry (PDB-MS) identified the nuclear poly(A)-binding protein ZC3H14 as a potential PABPN1-associated protein [40]. To confirm the association between PABPN1 and ZC3H14, we analyzed ZC3H14-proximal proteins in living human cells by PDB-MS (BioID) using a stable HEK293T cell line that conditionally expresses ZC3H14 fused to the BirA* biotin ligase. As a positive control, we analyzed a cell line that expresses BirA*-PABPN1 in parallel. To identify a list of ZC3H14-proximal proteins, samples from BirA*-ZC3H14 and control cells were analyzed by MS from five independent experiments. Significantly enriched factors were determined using Significance Analysis of INTeractome (SAINT), which is a statistical method for probabilistically scoring protein-protein interactions from MS data [43]. Enforcing a Bayesian false discovery rate ≤ 0.05 and Log_2_ fold-change ≥ 1.0, our analysis identified 75 and 138 ZC3H14- and PABPN1-proximal proteins, respectively (Fig. 1A and B and Supplementary Tables S3 and S4). ZC3H14 was among the high-confidence PABPN1-proximal proteins (Fig. 1B), consistent with our previous results [40]. Reciprocally, we identified PABPN1 among the ZC3H14-associated proteins (Fig. 1A). Significantly, of those 75 ZC3H14-proximal proteins, 62 (83%) were also identified as PABPN1-associated proteins (*P* < 2.5e-126) (Fig. 1C and Supplementary Table S5). Gene ontology (GO) analysis using ShinyGO [44] of those common 62 ZC3H14- and PABPN1-proximal proteins revealed significant enrichment in biological processes that were mainly associated with RNA splicing and nuclear export of mRNAs (Fig. 1D and E and Supplementary Table S6). The PBD-MS assays of ZC3H14 and PABPN1 further identified RBM26, RBM27, and ZFC3H1, proteins connected to poly(A) tail exosome targeting (PAXT) [15, 17] and ZC3H18, which has been shown to control PAXT connection with the nuclear cap binding complex [45] (Fig. 1E). Together, our PDB-MS assays reveal a physical connection between ZC3H14 and PABPN1, as well as a set of common associated proteins involved in splicing, nuclear export, and PAXT-mediated RNA decay.

We next validated the association between ZC3H14 and PABPN1 by affinity purification assays. We first confirmed the presence of endogenous ZC3H14 in eluates of GFP immunoprecipitates prepared from extracts of cells that conditionally expressed GFP-PABPN1 (Fig. 1F, lane 6). In contrast, control immunoprecipitations using extracts of cells that expressed GFP alone did not recover ZC3H14 (Fig. 1F, lane 2). Additionally, we examined whether the copurification of PABPN1 and ZC3H14 was dependent on RNA. Two different RNase conditions were used: (i) a mix of RNases A and T1, which catalyzes cleavage at pyrimidines (C and U for RNase A) and guanines (RNase T1), and (ii) RNase I, which hydrolyzes the phosphodiester bond of all four bases in RNA. Based on this base specificity, RNases A/T1 should not target poly(A) tails, whereas poly(A) tails are expected to be degraded by RNase I digestion. As shown in Fig. 1G, pre-treatment of total cell extracts with RNases A/T1 or RNase I resulted in robust digestion of cellular RNAs (compare lane 1–2 and 5–6 to lanes 3 and 7). Interestingly, whereas the copurification of ZC3H14 with PABPN1 was insensitive to RNases A/T1 digestion (Fig. 1F, compare lanes 6–7), the ZC3H14-PABPN1 copurification was reproducibly sensitive to RNAse I treatment (compare lane 8 to lane 6). Similar results were obtained by applying the RNase treatment to immunopurified complexes on beads (Supplementary Fig. S1). These affinity purification assays therefore (i) confirm the PABPN1-ZC3H14 proximity disclosed from our BioID assays and (ii) suggest that the association between PABPN1 and ZC3H14 depends on poly(A) tail integrity, supporting a view that these nuclear PABPs can share binding on poly(A) tails.

### PABPN1 levels influence the protein interaction network of ZC3H14 and ZC3H14 is important for the steady state accumulation of PABPN1 into nuclear speckles

**Figure 2.**
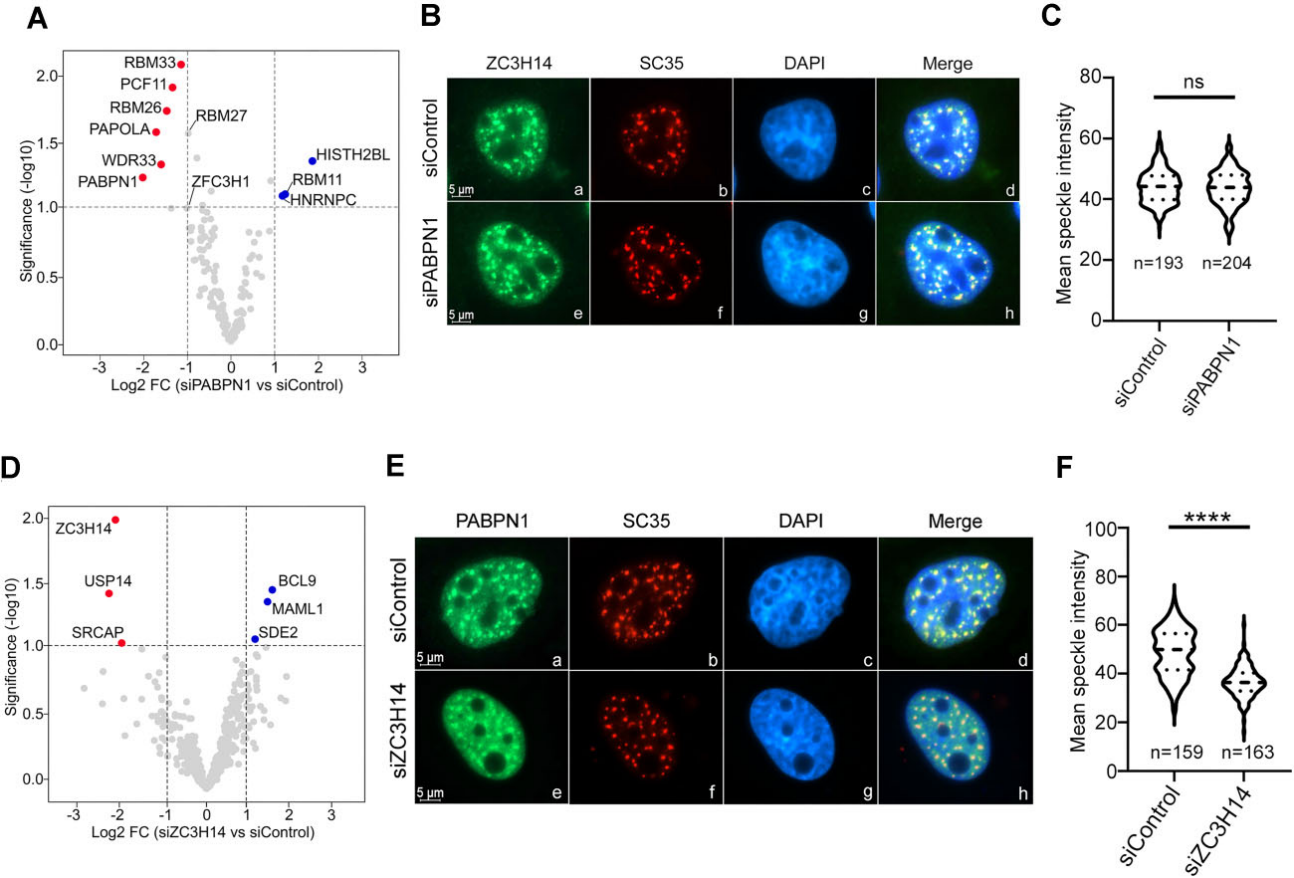
Functional connection between PABPN1 and ZC3H14. (**A**) Volcano plot of statistical significance (false discovery rate) against fold change (in log2) of ZC3H14-proximal proteins in HEK293T cells treated with PABPN1-specific siRNAs relative to cells treated with nontarget control siRNAs (*N* = 3 biological replicates). The dashed lines represent the thresholds for calling significant differential ZC3H14 association: FDR ≤ 0.05 and absolute log2 fold change ≥ 1.0. Proteins showing significantly decreased and increased ZC3H14-proximal biotinylation in PABPN1-deficient cells are shown in red and blue, respectively. (**B**) Immunostaining analysis of fixed and permeabilized HeLa cells that were previously transfected with control (a–d) and PABPN1-specific (e–h) siRNAs were stained with antibodies specific for ZC3H14 (a and e) and SC35 (b and f). DNA was stained with DAPI to show the nucleus (c and g). Images a–c, and e–g were merged to form d and h, respectively. Bar, 5 μm. (**C**) Violin plots with nuclear speckle ZC3H14 fluorescence mean intensity scores for cells transfected with the indicated siRNAs from two independent replicates. The total number of nuclei analyzed are indicated under each plot. Statistical differences were calculated using a unpaired student T-test for pairwise comparison (siControl and siPABPN1). ns, not significant. (**D**) Volcano plot of statistical significance (false discovery rate) against fold change (in log2) of PABPN1-proximal proteins in HEK293T cells treated with ZC3H14-specific siRNAs relative to cells treated with non-target control siRNAs (*N* = 3 biological replicates). The dashed lines represent the thresholds for calling significant differential PABPN1 association: FDR ≤ 0.05 and absolute log2 fold change ≥ 1.0. Proteins showing significantly decreased and increased PABPN1-proximal biotinylation in ZC3H14-deficient cells are shown in red and blue, respectively. (**E**) Immunostaining analysis of fixed and permeabilized HeLa cells that were previously transfected with control (a–d) and ZC3H14-specific (e–h) siRNAs were stained with antibodies specific for PABPN1 (a and e) and SC35 (b and f). DNA was stained with DAPI to show the nucleus (c and g). Images a–c, and e–g were merged to form d and h, respectively. Bar, 5 μm. (**F**) Violin plots with nuclear speckle PABPN1 fluorescence mean intensity scores for cells transfected with the indicated siRNAs from two independent replicates. The total number of nuclei analyzed are indicated under each plot. Statistical differences were calculated using a unpaired student T-test for pairwise comparison (siControl and siZC3H14). *P*-value ****, <0.0001.

The identification of a collection of RNA-binding proteins proximal to both ZC3H14 and PABPN1 (Fig. 1) prompted us to test whether a set of ZC3H14-associated proteins were dependent on PABPN1, and vice versa. To identify ZC3H14-associated proteins that depend on PABPN1 cellular levels, we used PDB-MS to analyze the proximal ZC3H14 interactome in HEK293T cells previously treated with PABPN1-specific and control siRNAs. We reproducibly achieved greater than 85% depletion of total PABPN1 levels for our ZC3H14 PDB-MS assays using HEK293T cells (Supplementary Fig. S2A). To identify proteins showing differential enrichment following PDB-MS analysis of ZC3H14, we used a statistical method (DEqMS) specifically developed for differential protein expression analysis using label-free quantification of MS data [46]. As shown in Fig. 2A (Supplementary Table S7), nine proteins showed significant changes in enrichment levels following ZC3H14 PDB-MS assays in PABPN1-deficient cells compared to control cells, with six and three proteins demonstrating decreased and increased enrichments, respectively. We focused on the set of significantly decreased interactions since none of the three identified proteins showing increased ZC3H14 association in PABPN1-deficient cells (HISTH2BL, RBM11, and HNRNPC) were among the identified ZC3H14 and PABPN1 interactome (Fig. 1). Gratifyingly, PABPN1 was among the proteins showing significantly decreased ZC3H14-dependent biotinylation in PABPN1-depleted cells (Fig. 2A), as expected from the siRNA-mediated knockdown. Next, of the 5 additional proteins showing significantly decreased association with ZC3H14 upon PABPN1 knockdown, 3 were among the set of the 62 interactions common to both ZC3H14 and PABPN1 (Fig. 2A): these are the PAXT component RBM26, the mRNA 3′ end processing factor PCF11, and lncRNA nuclear export factor RBM33. Analysis of PCF11 and RBM26 abundance using specific antibodies did not reveal major changes in PABPN1-deficient cells compared to control cells (Supplementary Fig. S2B), arguing that the reduction of ZC3H14-dependent biotinylation (Fig. 2A) is the result of reduced association rather than reduced levels of proximal proteins. In addition to RBM26, PAXT-connected proteins RBM27 and ZFC3H1 also showed reduced ZC3H14 proximity in PABPN1-deficient cells, nearly surpassing the cutoff established for statistical significance (Fig. 2A and Supplementary Table S7). Interestingly, the poly(A) polymerase alpha (PAPOLA) and the 3′ end processing factor, WDR33, also showed significantly decrease association with ZC3H14 in PABPN1-deficient cells (Fig. 2A). Whereas PAPOLA and WDR33 were among the proteins significantly enriched in PABPN1 PDB-MS assays (Fig. 1B and Supplementary Table S4), they did not pass the cut-off established by SAINT for the PDB-MS analysis of ZC3H14. We note, however, that PAPOLA and WDR33 were identified in three out of the five independent ZC3H14 PDB-MS assays with SAINT scores of 0.60 and 0.40, respectively (Supplementary Table S3). These results therefore identify a set of proteins involved in cleavage and polyadenylation (PCF11, WDR33, and PAPOLA), RNA export (RBM33), and polyadenylation-dependent RNA decay (RBM26, RBM27, and ZFC3H1) whose association with ZC3H14 is controlled by PABPN1 cellular levels.

We also examined whether the subcellular localization of ZC3H14 was affected by knocking down PABPN1 levels. We thus used HeLa cells for the immunofluorescence analysis of ZC3H14, because their large nucleus and flat morphology facilitate imaging and quantification. Indirect immunofluorescence of endogenous ZC3H14 in HeLa cells produced punctate signals that were restricted to the nucleus (Fig. 2B, panel a). This signal was substantially reduced in ZC3H14-depleted cells (see Supplementary Fig. S3A), confirming antibody specificity. To assess whether this punctate ZC3H14 signal corresponded to nuclear speckles, the anti-ZC3H14 staining was combined with an immunostaining for the speckle marker SC35. Comparison of the different immunostainings showed that ZC3H14 was concentrated in nuclear regions that colocalized with anti-SC35 staining (Fig. 2B, panels a–d). Immunostaining of ZC3H14 in PABPN1-deficient cells displayed similar punctate signals that largely colocalized with SC35 immunostaining (Fig. 2B, panels e–h). Quantification of the ZC3H14 signal intensities in nuclear speckles of PABPN1-depleted and control cells revealed similar fluorescence (Fig. 2C). We conclude that PABPN1 controls the network of proteins proximal to ZC3H14 but does not affect ZC3H14 enrichment into nuclear speckles.

We next examined whether knocking down cellular levels of ZC3H14 affected the protein interaction network of PABPN1. We therefore analyzed PABPN1-proximal proteins by PBD-MS in control and ZC3H14-deficient cells (Supplementary Fig. S4). In total, we identified six proteins showing significantly different enrichments in PDB-MS assays of PABPN1 using cells depleted of ZC3H14 (Fig. 2D; Supplementary Table S8). Of those six differentially enriched proteins, only ZC3H14 was among the significant PABPN1 proximal proteins identified by PDB-MS (Fig. 1B and Supplementary Table S2). Yet, we note that SDE2 could be a potentially interesting candidate, as this protein was recently shown to be an RNA-binding protein important for pre-mRNA splicing [47].

Next, we examined the impact of ZC3H14 deficiency on the subcellular distribution of PABPN1 using an antibody specific for endogenous PABPN1 (Supplementary Fig. S3B). As shown in Fig. 2E (panels a–d), endogenous PABPN1 primarily localized in the nucleus of HeLa cells with enrichment in nuclear speckles, consistent with previous observations [48,49]. Notably, analysis of PABPN1 immunostaining in ZC3H14-depleted cells revealed a reduction in fluorescence signal in nuclear speckles together with more diffuse PABPN1 signal in the nucleoplasm (Fig. 2E, panels e–h and Supplementary Fig. S5). Quantification of PABPN1 fluorescence intensities in nuclear speckles confirmed a significant decrease in the steady state accumulation of PABPN1 in nuclear speckles of HeLa cells when ZC3H14 levels are reduced (Fig. 2F). Collectively, the protein interaction assays and the localization studies shown in Fig. 2 support the idea of a functional connection between the nuclear poly(A)-binding proteins ZC3H14 and PABPN1.

### Antagonistic roles of ZC3H14 and PABPN1 in the regulation of the snoRNA host gene *SNHG19*

**Figure 3.**
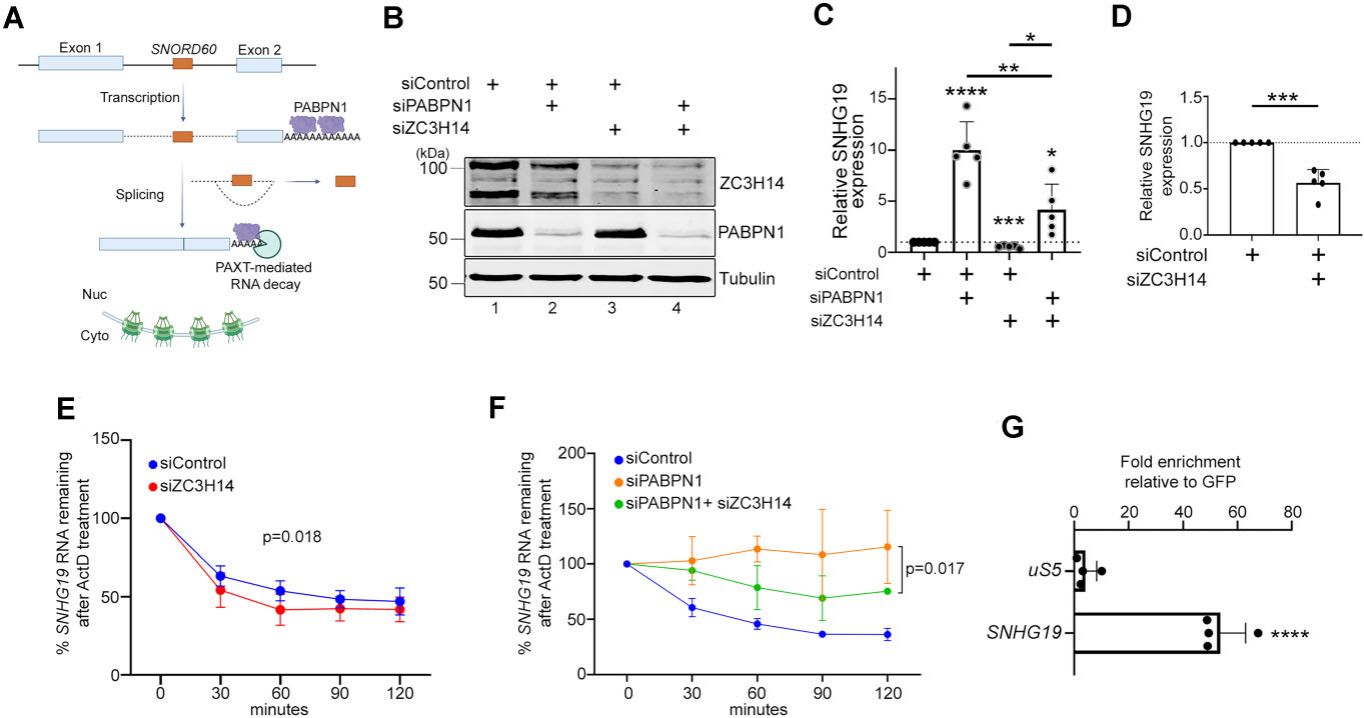
PABPN1 and ZC3H14 antagonistically regulate the expression of the *SNHG19* lncRNA. (**A**) Schematic of the *SNHG19* lncRNA expression. The blue rectangles represent the *SNHG19* non-coding exons, while the red box corresponds to intronic *SNORD60*. Polyadenylation-dependent RNA degradation of the spliced *SNHG19* lncRNA is promoted by PABPN1 and PAXT in the nucleus. (**B**) Western blot analysis of ZC3H14, PABPN1, and tubulin proteins using total extracts prepared from HeLa cells previously transfected with the indicated siRNAs. The antibodies used for Western blot analysis are shown on the right and molecular weights markers on the left. (**C** and **D**) RT-qPCR analysis of *SNHG19* lncRNA from total RNA harvested from HeLa cells that were previously transfected with the indicated siRNAs. Data and error bars represent the means and standard deviations of five independent experiments, respectively. *P*-values (∗) ≤0.05; (∗∗) ≤0.01; (∗∗∗) ≤0.001; (∗∗∗∗) ≤0.0001 were determined with an unpaired Student t test. (**E** and **F**) HeLa cells previously transfected with the indicated siRNAs were treated with 5 mg/ml actinomycin D and total RNA was isolated at the indicated time points. The degradation rate of spliced *SNHG19* lncRNA was determined by RT-qPCR analysis and normalized to *18S* rRNA. Data and error bars represent the mean and standard deviation of three independent experiments. *P*-values were calculated using Two-way ANOVA tests. (**G**) Fold mRNA enrichment (IP/input ratio) for the indicated the *uS5* mRNA and the *SNHG19* lncRNA in GFP-ZC3H14 immunoprecipitates was analyzed by RT-qPCR and normalized to a control housekeeping mRNA (*GAPDH*). Values were then set to 1.0 for the control GFP purification. Data and error bars represent the means and standard deviations of four independent experiments, respectively. *P* value (∗∗∗∗) <0.0001 was determined with an unpaired Student t test.

To get further insights into the functional significance of the ZC3H14 and PABPN1 association, we analyzed the expression of a known target of PABPN1 and PAXT, the snoRNA host gene 19 (*SNHG19*). *SNHG19* is located on human chromosome 16 and expresses a 564 nt-long primary transcript that includes a 170 nt-long intron, from which the C/D box snoRNA SNORD60 is produced after splicing and debranching (Fig. 3A). The spliced transcript from the two *SNHG19* exons produces a lncRNA that is targeted by polyadenylation-mediated RNA decay via PABPN1 and PAXT (Fig. 3A). The *SNHG19* lncRNA is in fact one of the transcripts with the strongest accumulation in PABPN1- and PAXT-deficient cells [13, 15]. To test if ZC3H14 contributed to PABPN1-mediated RNA decay, we performed single and double depletions of ZC3H14 and PABPN1 in HeLa cells (Fig. 3B) and analyzed *SNHG19* expression by RT-qPCR. Consistent with previous studies [13, 15], the knockdown of PABPN1 resulted in a 10-fold increase in *SNHG19* lncRNA levels (Fig. 3C). In contrast, a significant 2-fold reduction in *SNHG19* lncRNA levels was found in ZC3H14-deficient cells (Fig. 3C–D). Notably, the co-depletion of both ZC3H14 and PABPN1 showed a significant decrease in *SNHG19* lncRNA compared to the single depletion of PABPN1 (Fig. 3C). These results suggest that ZC3H14 and PABPN1 have opposing roles in PAXT-mediated nuclear degradation of the *SNHG19* lncRNA.

We have previously demonstrated that the increased level of *SNHG19* lncRNA in PABPN1-deficient cells is the consequence of increased RNA stability [13]. To assess whether the reduction in *SNHG19* lncRNA abundance in cells deficient for ZC3H14 is the result of increased RNA turnover, actinomycin D was used to inhibit RNAPII transcription in ZC3H14-depleted and control cells. RNA decay of *SNHG19* was then followed over time by RT-qPCR and data were normalized using the *18S* rRNA. Depletion of ZC3H14 significantly enhanced the turnover of the *SNHG19* lncRNA (Fig. 3E), consistent with its downregulation in ZC3H14-deficient cells. As a negative control, the stability of the *myc* mRNA was not changed in the same ZC3H14 knockdown conditions (Supplementary Fig. S6). We also compared the stability of the *SNHG19* lncRNA between single PABPN1 and double PABPN1 + ZC3H14 depletions. As shown in Fig. 3F, the co-depletion of ZC3H14 and PABPN1 destabilized the *SNHG19* lncRNA compared to the single PABPN1 depletion (compare green to orange curve). We thus conclude that whereas PABPN1 promotes PAXT-mediated turnover of the *SNHG19* lncRNA, ZC3H14 promotes its stability.

We next addressed whether ZC3H14 directly regulates the expression of the *SNHG19* lncRNA. To test this, RIP assays were used to determine whether ZC3H14 specifically binds to *SNHG19*. We therefore affinity purified GFP and GFP-ZC3H14 from human cell extracts, and RNA was subsequently isolated from anti-GFP precipitates and analyzed by RT-qPCR. The data were normalized to the *GAPDH* mRNA to control for experimental variation, and the values were set to 1.0 for the control GFP purification. As shown in Fig. 3G, we observed a significant enrichment of the *SNHG19* lncRNA in GFP-ZC3H14 precipitates relative to the GFP control. Importantly, the copurification of the *SNHG19* lncRNA with GFP-ZC3H14 was specific, as a control transcript (the *uS5* mRNA) was not selectively enriched with GFP-ZC3H14 (Fig. 3G). These data support a model, whereby ZC3H14 directly controls PABPN1-dependent decay of the *SNHG19* lncRNA.

### ZC3H14 restricts PAXT-dependent RNA degradation

**Figure 4.**
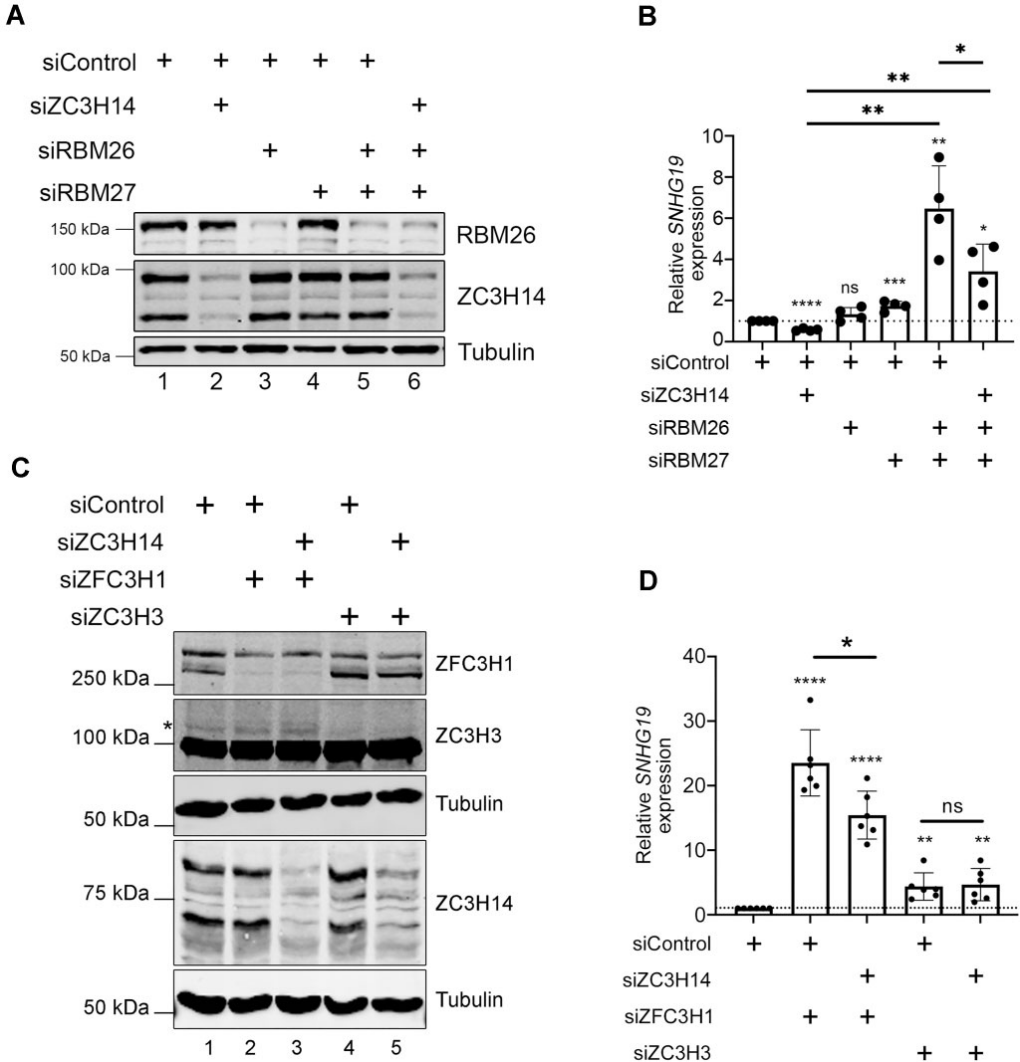
ZC3H14 restricts PAXT-mediated degradation of the *SNHG19* lncRNA. (**A**) Western blot analysis of ZC3H14, RBM26, and tubulin proteins using total extracts prepared from HeLa cells previously transfected with the indicated combination of siRNAs. The antibodies used for Western blot analysis are shown on the right and molecular weights markers on the left. (**B**) RT-qPCR analysis of *SNHG19* lncRNA from total RNA harvested from HeLa cells that were previously transfected with the indicated combination of siRNAs. Data and error bars represent the means and standard deviations of four independent experiments, respectively. *P*-values (∗) ≤0.05; (∗∗) ≤0.01; (∗∗∗) ≤0.001; (∗∗∗∗) ≤0.0001 were determined with an unpaired Student t test. (**C**) Western blot analysis of ZC3H14, ZFC3H1, ZC3H3, and tubulin proteins using total extracts prepared from HeLa cells previously transfected with the indicated combination of siRNAs. The antibodies used for Western blot analysis are shown on the right and molecular weights markers on the left. The asterisk (*) indicates the position of ZC3H3. (**D**) RT-qPCR analysis of *SNHG19* lncRNA from total RNA harvested from HeLa cells that were previously transfected with the indicated combination of siRNAs. Data and error bars represent the means and standard deviations of six independent experiments, respectively. *P*-values (∗) ≤0.05; (∗∗) ≤0.01; (∗∗∗∗) ≤0.0001 were determined with an unpaired Student t test.

PABPN1 is functionally connected to the Poly(A) Tail eXosome Targeting (PAXT) network. In addition to PABPN1, our PDB-MS analysis disclosed the PAXT-associated RNA-binding proteins, RBM26, RBM27, and ZFC3H1 as ZC3H14-proximal proteins (Fig. 1). We therefore examined whether the antagonistic role of ZC3H14 in restricting PABPN1-dependent decay of the *SNHG19* lncRNA was also observed after knockdown of additional PAXT cofactors. Whereas individual depletions of RBM26 and RBM27 did not result in a substantial increase in *SNHG19* lncRNA, co-depletion of RBM26 and RBM27 resulted in a 7-fold increase in *SNHG19* levels (Fig. 4A and B and Supplementary Fig. S7). These data are consistent with the redundant role of RBM26 and RBM27 in PAXT-mediated RNA decay [17]. Next, we examined the impact of knocking down ZC3H14 in cells deficient for both RBM26 and RBM27 (Fig. 4A and Supplementary Fig. S7). As shown in Fig. 4B, the depletion of ZC3H14 in RBM26/RBM27-deficient cells significantly decreased the steady state accumulation of *SNHG19* lncRNA caused by deficiencies of both RBM26 and RBM27. Similarly to results obtained with PABPN1 (Fig. 3), these data indicate that ZC3H14 functions in opposition to RBM26/RBM27-dependent RNA decay.

We also examined whether ZC3H14 restricts the activity of ZFC3H1 and ZC3H3 in connection to PAXT-dependent RNA decay by co-depletion of either PAXT cofactors with ZC3H14 (Fig. 4C). As shown in Fig. 4D, single depletions of ZFC3H1 or ZC3H3 resulted in a significant increase of *SNHG19* lncRNA levels, consistent with previous findings [17, 50]. As shown for PABPN1 and RBM26/RBM27, the absence of ZC3H14 in ZFC3H1-depleted cells significantly reduced the accumulation of the *SNHG19* lncRNA compared to the single depletion of ZFC3H1 (Fig. 4D). Interestingly, this was not the case for the co-depletion of ZC3H3 and ZC3H14, which showed no significant difference compared to the ZC3H3 single depletion (Fig. 4D). Thus, given the involvement of PABPN1, RBM26, RBM27, and ZFC3H1 in PAXT-mediated RNA decay, our data indicate that ZC3H14 restricts PAXT activity on selected targets.

### A set of non-coding RNA genes are co-regulated by ZC3H14 and PABPN1

To obtain a broader perspective of genes that are antagonistically regulated by PABPN1 and ZC3H14, we analyzed total RNA prepared from HeLa cells depleted for PABPN1, ZC3H14, and PABPN1 + ZC3H14 by strand specific RNA-seq. After filtering for genes expressed ≥0.5 counts per million (cpm), 1223 and 1293 genes were upregulated and downregulated, respectively, by more than 1.5-fold in PABPN1-deficient cells (Supplementary Table S9). A small number of genes was impacted upon depletion of ZC3H14: 362 and 379 transcripts that were upregulated and downregulated, respectively, by more than 1.5-fold (Supplementary Table S9). Our RNA-seq data are consistent with previous transcriptome-wide analyses that revealed that a ZC3H14 deficiency shows very few significant expression changes [34, 51]. We also examined total poly(A) RNA distribution by oligo d(T) fluorescent *in situ* hybridization (FISH) in ZC3H14-depleted HeLa cells, which was essentially identical to control cells (Supplementary Fig. S8), as similarly reported using Neuro 2A cells [32]. Collectively, these data suggest that ZC3H14 does not globally contribute to RNA export or RNA stability.

**Figure 5.**
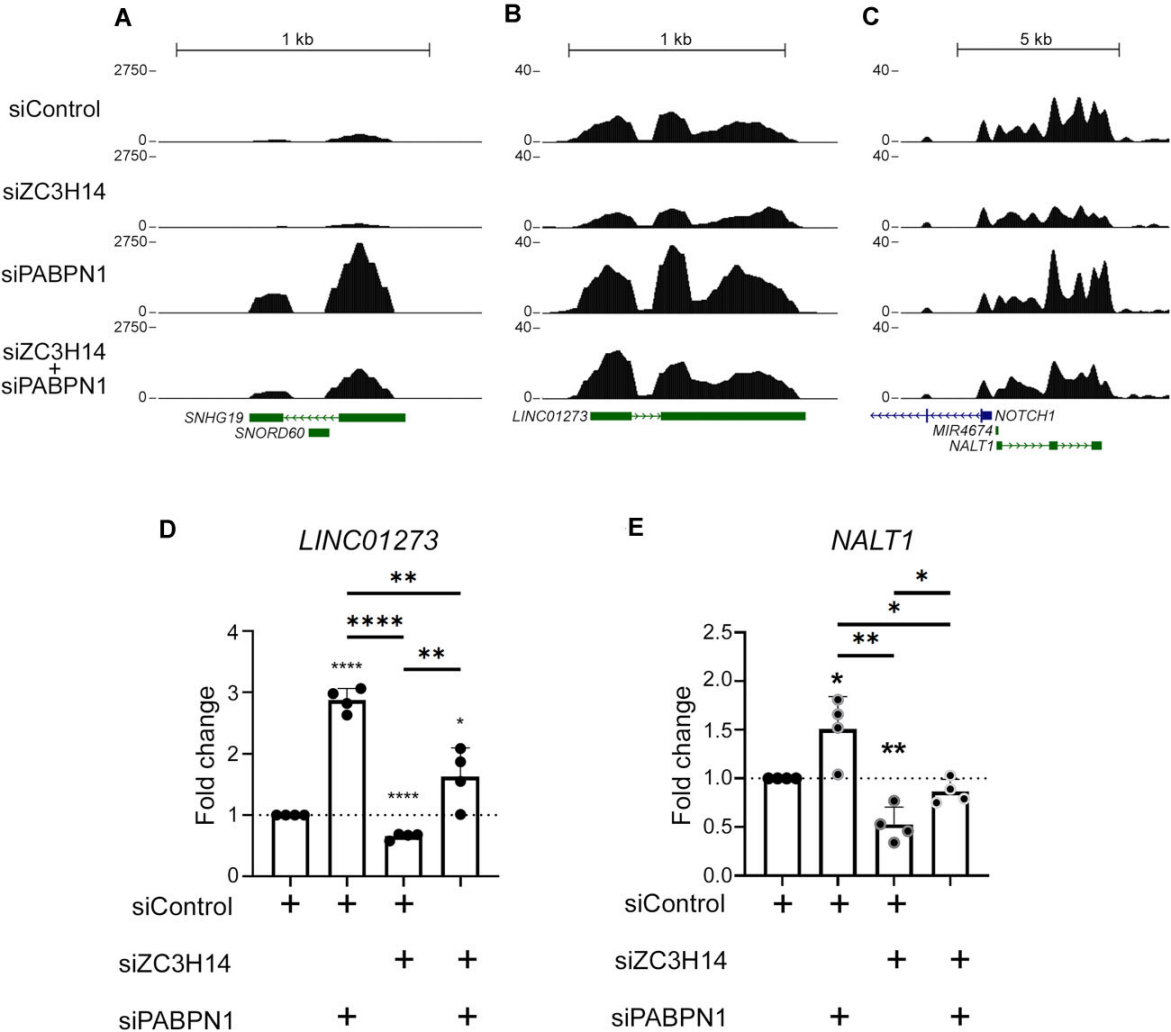
Antagonistic regulation of ncRNA expression by PABPN1 and ZC3H14. (**A**–**C**) Read coverage over the *SNHG19* (**A**), *LINC01273* (**B**), and *NALT1* (**C**) genes from RNA-seq data of HeLa cells previously transfected with the indicated siRNAs. Transcript annotations are from GENCODE V44. (**D**–**E**) RT-qPCR analysis of *LINC01273* (**D**) and *NALT1* (**E**) ncRNA genes using total RNA harvested from HeLa cells that were previously transfected with combination of the indicated siRNAs. Data and error bars represent the means and standard deviations of five independent experiments, respectively. *P*-values (∗) ≤0.05; (∗∗) ≤0.01; (∗∗∗) ≤0.001; (∗∗∗∗) ≤0.0001 were determined with an unpaired Student t test.

We next compared the set of 1223 genes upregulated by more than 1.5-fold in PABPN1-deficient cells to the 379 genes downregulated by more than 1.5-fold in ZC3H14-deficient cells, and found a significant overlap of 39 genes that were antagonistically regulated by PABPN1 and ZC3H14 (*P*< 1.06–05; Fisher's exact test). Notably, of those 39 antagonistically-regulated genes, 26 (67%) code for either lncRNAs, antisense RNAs, or novel non-coding transcripts (Supplementary Table S10). As expected from the aforementioned results (Fig. 3), the *SNHG19* lncRNA was among the list of 39 genes antagonistically-regulated by PABPN1 and ZC3H14 (Fig. 5A). We also validated the opposing roles of PABPN1 and ZC3H14 on the regulation of two additional lncRNA genes, *LINC01273* (Fig. 5B) and *NALT1* (Fig. 5C), by RT-qPCR using total RNA from independent siRNA-mediated depletion experiments (Fig. 5D and E). These data suggest that PABPN1 and ZC3H14 negatively and positively control, respectively, the expression of a set of lncRNAs via PAXT-mediated RNA decay.

### PABPN1 and ZC3H14 control *SNHG19* lncRNA nuclear retention

**Figure 6.**
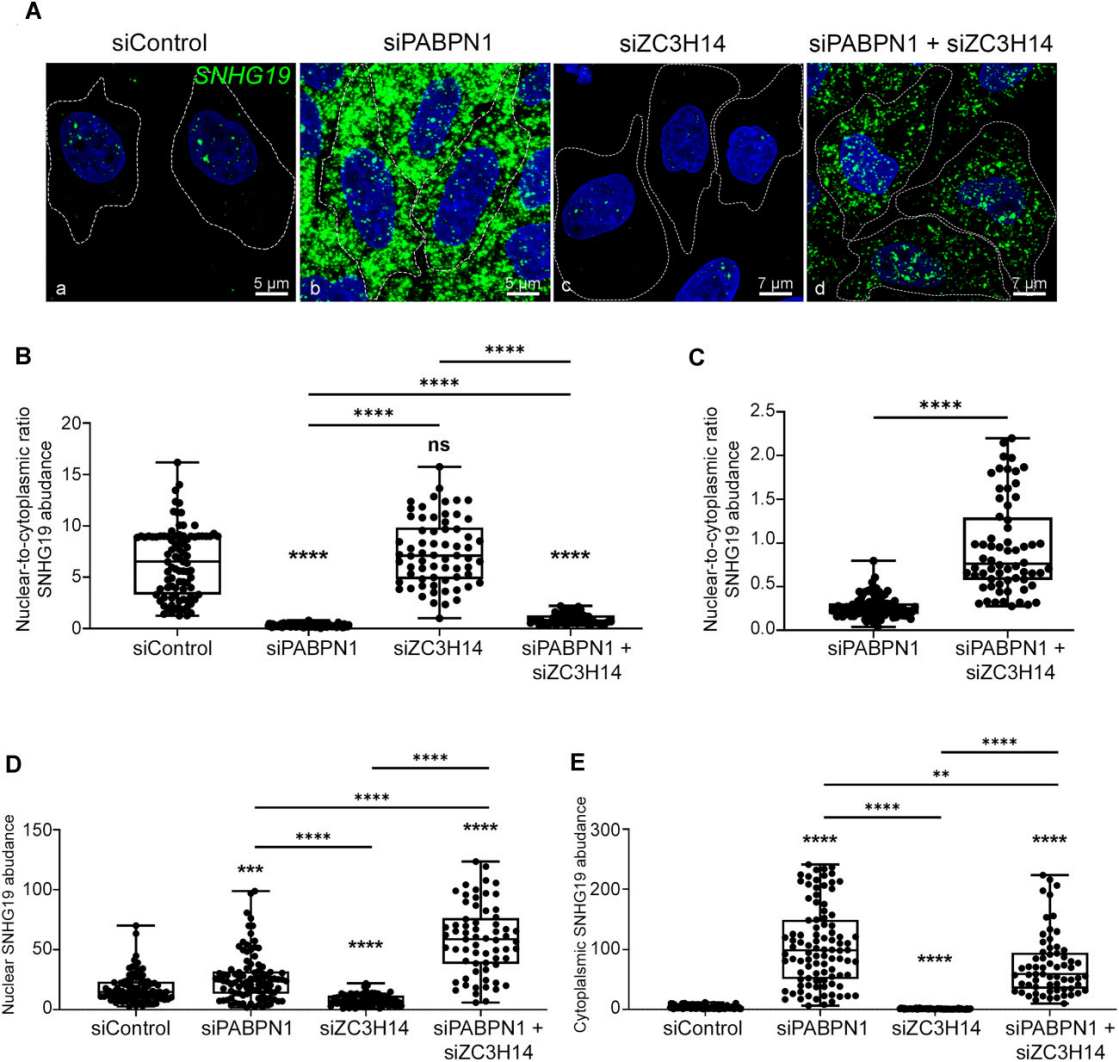
*SNHG19* lncRNA nuclear retention is controlled by PABPN1 and ZC3H14. (**A**) Representative images of RNAscope *in situ* hybridization showing the localization of the *SNHG19* lncRNA (green) in HeLa cells that were previously transfected with the indicated siRNAs. (**B**) Box plot showing the nuclear-to-cytoplasmic intensity ratio of *SNHG19* signal for cells transfected with the indicated siRNAs. Data are from two independent experiments. In total, 91, 97, 67, and 65 cells were quantified for siControl, siPABPN1, siZC3H14, and the co-depletion of PABPN1 and ZC3H14 from two independent FISH experiments. (**C**) Box plot showing the nuclear-to-cytoplasmic intensity ratio of *SNHG19* signal of cells lacking PABPN1 compared to cells deficient for both PABPN1 and ZC3H14. (**D** and **E**) Box plot showing the nuclear (**D**) and cytoplasmic (**E**) intensity of *SNHG19* signal for cells transfected with the indicated siRNAs. ns, *P* value > 0.05; (∗∗), <0.01; (∗∗∗), <0.001; (∗∗∗∗),and <0.0001 were determined with an unpaired Student t test.

The underlying mechanism by which PABPN1 facilitates PAXT-mediated nuclear RNA decay remains elusive. Recently, we showed that PABPN1 prevents the premature export of a unspliced viral reporter transcript from the nucleus [40]. To assess whether PABPN1 promotes PAXT-mediated RNA decay of the *SNHG19* lncRNA by impeding its nuclear export, we performed RNAscope *in situ* hybridization using probes spanning *SNHG19* exonic sequences to visualize its subcellular localization. In HeLa cells transfected with a nontarget control siRNA, the *SNHG19* lncRNA was localized in both the nucleus and cytoplasm (Fig. 6A, panel a), but was generally more abundant in the nuclear compartment (Fig. 6B). Strikingly, the depletion of PABPN1 resulted in a significant increase in *SNHG19* lncRNA signal in the cytoplasm (Fig.6A, panel b; Fig. 6E) as well as a small, yet significant increase in the nucleus (Fig. 6D). We thus conclude that the robust increase of *SNHG19* lncRNA levels detected by RT-qPCR and RNA-seq in PABPN1-deficient cells [13, 15] primarily represents cytoplasmic accumulation. In contrast, ZC3H14 loss-of-function resulted in decreased *SNHG19* lncRNA signal in both the nucleus (Fig. 6A, panel c and Fig. 6D) and the cytoplasm Fig. 6E). This FISH analysis suggest that the absence of ZC3H14 impairs nuclear export of the *SNHG19* lncRNA, thereby promoting PAXT-dependent nuclear decay, consistent with our RT-qPCR (Figs 3–4) and RNA-seq (Fig. 5) data. Interestingly, the co-depletion of ZC3H14 and PABPN1 significantly increased the nuclear-to-cytoplasmic ratio of the *SNHG19* lncRNA compared to the single depletion of PABPN1 (Fig. 6A, compare panels b and d; Fig. 6C). This increase in nuclear-to-cytoplasmic ratio was the result of both decreased cytoplasmic signal (Fig. 6E) and increased nuclear signal (Fig. 6D) compared to the individual depletion of PABPN1. We also used cellular fractionation assays (Supplementary Fig. S9A) to independently validate the cytoplasmic accumulation of *SNHG19* lncRNA in PABPN1-deficient cells and examine if this accumulation preferentially affected the spliced or unspliced isoform of *SNHG19*. Notably, RT-qPCR analysis of cytoplasmic and nuclear RNA revealed that it is mainly the spliced isoform of *SNHG19* that accumulates in the cytoplasm upon PABPN1 depletion, whereas cytoplasmic levels of the unspliced isoform did not significantly change in PABPN1-deficient cells (Supplementary Fig. S9B). Collectively, our results suggest that PABPN1 contributes to PAXT-mediated RNA decay by retaining polyadenylated lncRNAs in the nucleus for degradation by the RNA exosome, whereas ZC3H14 appears to function in opposition to PABPN1 by stimulating nuclear export of spliced *SNHG19*.

**Figure 7.**
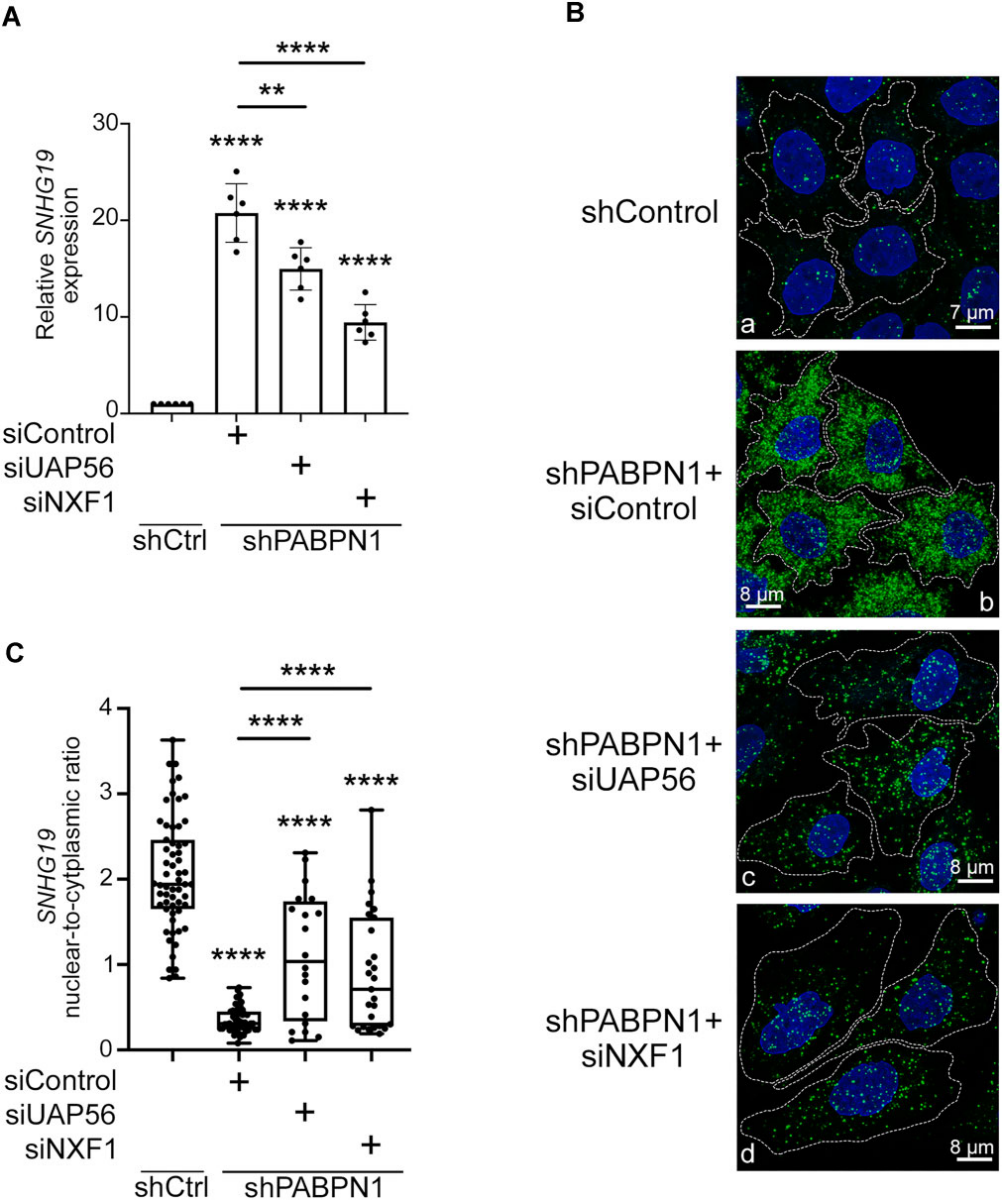
Reducing RNA export efficiency counters the cytoplasmic accumulation of *SNHG19* resulting from a PABPN1 deficiency. (**A**) RT-qPCR analysis of *SNHG19* using total RNA prepared from HeLa cells that conditionally express (+Doxycycline) a nontarget control shRNA (shCtrl) or a PABPN1-specific shRNA (shPABPN1) and that were previously transfected with siRNAs specific to UAP56, NXF1, or a nontarget control siRNA. Data and error bars represent the means and standard deviations of six independent experiments, respectively. (**B**) Representative images of RNAscope *in situ* hybridization showing the localization of the *SNHG19* lncRNA (green) in HeLa cells that conditionally express (+Doxycycline) a nontarget control shRNA (shCtrl) or a PABPN1-specific shRNA (shPABPN1) and that were previously transfected with the indicated siRNAs. (**C**) Box plot showing the nuclear-to-cytoplasmic intensity ratio of *SNHG19* signal for cells transfected with the indicated siRNAs. In total, 59, 52, 20, and 27 cells were quantified for shCtrl cells and the siControl, siUAP56, and siNXF1 in shPABPN1-induced cells, respectively, from two independent FISH experiments. (∗∗), *P*-value <0.01; (∗∗∗∗), *P* value <0.0001 were determined with an unpaired Student t test.

To get further evidence supporting that ZC3H14 promotes lncRNA stability in opposition to PABPN1 by assisting in the nuclear export of polyadenylated lncRNAs, we examined the impact of depleting proteins that act in RNA export in PABPN1-deficient cells. Based on findings indicating selective and redundant RNA export mechanisms [52], we used siRNA-mediated depletion to target the nuclear export factor NXF1 and the TREX component UAP56. Consistent with previous studies, a large proportion of polyadenylated transcripts was no longer exported after knockdown of NXF1 or UAP56, as demonstrated by the reduction of cytoplasmic poly(A) RNA signal using oligo d(T) FISH analysis (Supplementary Fig. S8). We next depleted NXF1 and UAP56 in PABPN1-deficient HeLa cells (Supplementary Fig. S10) and used RT-qPCR and FISH analysis to examine the steady state level and the subcellular distribution of the *SNHG19* lncRNA, respectively. As shown in Fig. 7A, knocking down NXF1 or UAP56 in PABPN1-deficient cells significantly reduced the accumulation of *SNHG19* lncRNA compared to the single PABPN1 depletion. The greater effect of NXF1 depletion compared to UAP56 on PABPN1-dependent *SNHG19* accumulation is consistent with NXF1 preferentially exporting transcripts with few exons [52]. Additionally, FISH analyses of *SNHG19* localization by RNAscope indicated that the co-depletion of NXF1 or UAP56 with PABPN1 generally increased the nuclear-to-cytoplasmic ratio of the *SNHG19* lncRNA compared to a single PABPN1 depletion, which showed mainly cytoplasmic *SNHG19* localization (Fig. 7B, compare panels c–d to b; quantifications shown in Fig. 7C). Thus, reducing RNA export efficiency in PABPN1-deficient cells resulted in effects on the *SNHG19* lncRNA that are similar to a ZC3H14 loss-of-function (Fig. 6), supporting a model where ZC3H14 promotes lncRNA stability in opposition to PABPN1 by reducing *SNGH19* nuclear residence time.

### lncRNA stabilization by PAXT deficiency does not necessarily result in cytoplasmic accumulation

**Figure 8.**
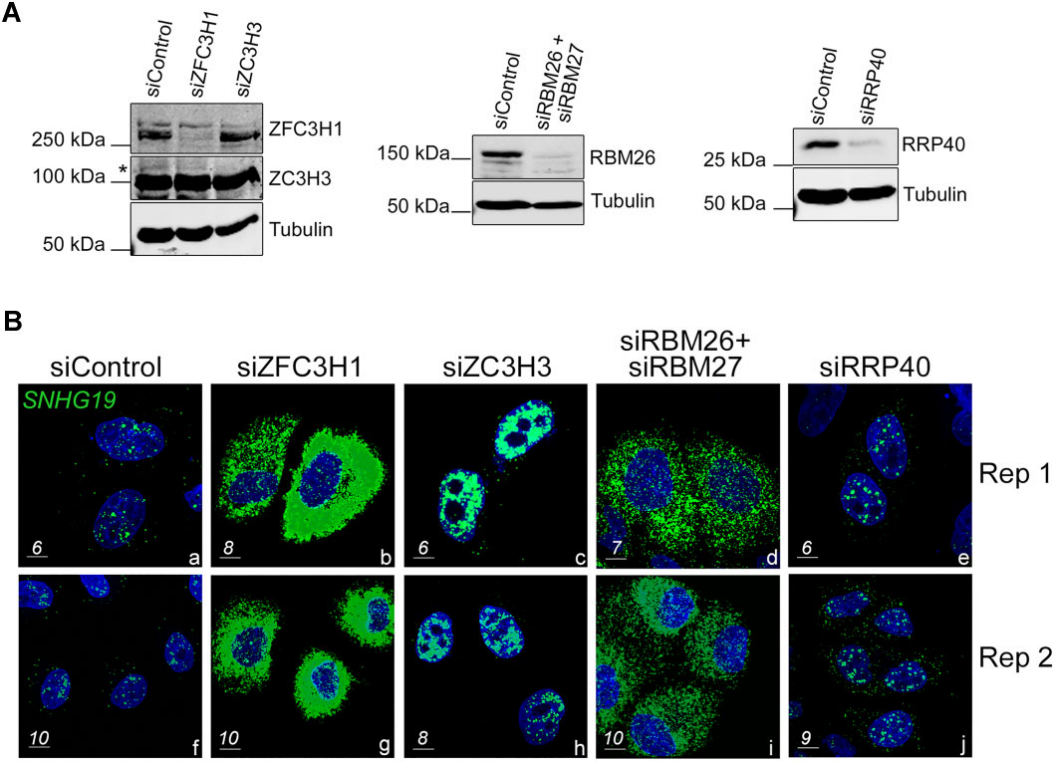
Inhibition of PAXT-dependent exosome-mediated RNA decay does not necessarily result in cytoplasmic RNA accumulation. (**A**) Western blot analysis of ZFC3H1 and ZC3H3 (left), RBM26 (center), and RRP40 (right) proteins using total extracts prepared from HeLa cells previously transfected with the indicated siRNAs. The antibodies used for Western blot analysis are shown on the right and molecular weights markers on the left. The asterisk (*) indicates the position of ZC3H3. (**B**) Representative images of RNAscope *in situ* hybridization from independent experiments (Rep 1 and 2) showing the localization of the *SNHG19* lncRNA (green) in HeLa cells that were previously transfected with siRNAs targeting ZFC3H1 (b, g), ZC3H3 (c, h), RBM26 and RBM27 (d, i), RRP40 (e, j), and control nontarget siRNAs (a, f). Scale bars (μm) are shown in lower left corner.

Our RNA localization studies suggest that PABPN1 contributes to PAXT-mediated RNA decay by retaining polyadenylated lncRNAs in the nucleus for degradation by the RNA exosome (Fig. 6-7). According to this model, polyadenylated PAXT targets would evade nuclear decay by bypassing nuclear retention in PABPN1-deficient cells, being actively exported into the cytoplasm. An alternative explanation, however, is that depletion of PABPN1 leads to strong nuclear accumulation of polyadenylated PAXT targets, and that these stabilized transcripts leak out of the nucleus via the nuclear pore complex. Although these mechanisms are not necessarily mutually exclusive, we tested whether abolishing PAXT-dependent exosome-mediated nuclear decay essentially results in the cytoplasmic accumulation of a stabilized target RNA. We therefore depleted PAXT components ZFC3H1, ZC3H3, and RBM26/RBM27 as well as the core RNA exosome subunit RRP40 in HeLa cells (Fig. 8A) and examined the subcellular distribution of the stabilized *SNHG19* lncRNA by FISH. As shown in Fig. 8B, stabilized *SNGH19* lncRNAs mostly accumulated in the cytoplasm of ZFC3H1-deficient cells (panels b and g), consistent with previous findings indicating that ZFC3H1 assists in the nuclear retention of PAXT target transcripts [16, 50]. Similarly, co-depletion of RBM26 and RBM27 showed cytoplasmic lncRNA accumulation (Fig. 8B, panels d and i). In striking contrast, the stabilized *SNHG19* lncRNA mainly accumulated in the nucleus of ZC3H3-deficient cells (Fig. 8B, panels c and h). Depletion of RNA exosome component RRP40 resulted in the accumulation of *SNGH19* in distinct nuclear foci (Fig. 8B, panels e and j), as previously reported [50]. There results therefore indicate that the stabilization of a PAXT target transcript does not necessarily result in cytoplasmic accumulation, arguing in favor for directs roles of PABPN1, ZFC3H1, and RBM26/27 in the nuclear retention of PAXT substrates.

## Discussion

We and others have previously reported that the nuclear poly(A)-binding protein PABPN1 functions in the degradation of nuclear lncRNAs via the RNA exosome [13–15]. Yet, the underlying mechanism by which PABPN1 promotes lncRNA turnover in the nucleus has remained largely elusive. We show here that PABPN1 contributes to the nuclear retention of target lncRNAs. In the absence of PABPN1, unstable polyadenylated lncRNAs such as the spliced product of a SNHG strongly accumulate in the cytoplasm, thereby escaping nuclear decay by the RNA exosome. Furthermore, we report that another nuclear PABP, the conserved ZC3H14 RNA-binding protein, functions antagonistically to PABPN1/PAXT in the control of nuclear lncRNA turnover.

Using proximity biotinylation to define the interactome of two nuclear poly(A)-binding proteins in living human cells, we disclose a comprehensive inventory of factors that associate with PABPN1 and ZC3H14. Our proximity map of PABPN1 and ZC3H14 revealed that both proteins copurify and associate with a similar set of RNA-related factors. The two major molecular functions that were associated with the common set of PABPN1/ZC3H14 interactors were RNA splicing and nuclear RNA export (Fig. 1). This protein interaction network is consistent with the localization of PABPN1 and ZC3H14 to nuclear speckles (Fig. 2), as many of the PABPN1/ZC3H14-proximal proteins are known to be enriched in nuclear speckles, including the TREX subunits ALYREF and CHTOP [53]. We also found that some of the strongest PABPN1/ZC3H14-dependent biotinylated proteins were components of the PAXT connection, including RBM26, RBM27, ZFC3H1, and the PAXT regulator ZC3H18 (Fig. 1). The connection of PABPN1 with PAXT was previously established [15,40]; yet, physical and functional connections between ZC3H14 and PAXT in the turnover of prematurely terminated RNAs (ptRNAs) were reported only recently [34]. Although our data and the findings reported by Insco et *al*. [34] both support physical associations between ZC3H14 and PAXT, they contrast on ZC3H14’s role in the regulation of nuclear RNA surveillance: negative control of PAXT-dependent decay of select lncRNAs (this study) versus positive regulation of ptRNA turnover [34]. We note, however, that siRNA-mediated depletion of ZC3H14 revealed few changes in ptRNA expression, whereas a phospho-mimetic version of ZC3H14 showed more impact on the expression of ptRNAs [34]. Consistent with results showing that the sole depletion of ZC3H14 does not substantially affect ptRNA expression [34], an unstable transcript resulting from premature cleavage and polyadenylation in the first intron of *PCF11* [54,55] was unaffected by ZC3H14 deficiency, but accumulated in PABPN1-deficient cells (Supplementary Fig. S11). It is therefore likely that the activity of ZC3H14 as a positive [34] and negative (our study) regulator of nuclear RNA turnover is controlled by phosphorylation.

We did not expect to find a key role for PABPN1 in RNA nuclear retention given that another protein of the PAXT connection, the zinc finger protein ZFC3H1, had previously been shown to prevent the transport of PAXT substrates to the cytoplasm [16,50]. Given that PABPN1 and ZFC3H1 share substrates for polyadenylation-dependent RNA decay via PAXT [13,15,16,50], our data indicate that the presence of ZFC3H1 is not sufficient for the nuclear retention of PAXT substrates and that poly(A)-bound PABPN1 is also required to retain target transcripts to the nucleus for exosome-mediated decay. It therefore appears that PABPN1 and the ZFC3H1-MTR4 dimer function cooperatively to prevent the nuclear export of target polyadenylated RNAs, a key step to sorting select transcripts destined for exosome-mediated nuclear decay via PAXT. Interestingly, we find that ZC3H3, a zinc finger protein that physically interacts with ZFC3H1 and is essential for PAXT-mediated nuclear RNA decay [17], is exclusively involved in RNA degradation in the nucleus, as demonstrated by the nuclear accumulation of *SNHG19* in ZC3H3-deficient cells (Fig. 8). Our results thus distinguish between the role of PAXT in contributing to RNA nuclear retention (PABPN1, ZFC3H1, and RBM26/RBM27) versus components exclusively involved in RNA degradation in the nucleus (ZC3H3 and exosome). The cytoplasmic accumulation of PAXT substrates in the absence of PABPN1 is also consistent with the idea that nuclear export kinetic is a key mechanism allowing transcripts to evade RNA decay by the nuclear exosome [50,56,57].

How does PABPN1 promote the retention of PAXT substrates into the nucleus? The simplest model is that PABPN1 facilitates loading of ZFC3H1 onto target transcripts, binding of which has been shown to functionally compete with the recruitment of the RNA export factor ALYREF [50,56]. Consistent with this model is evidence showing reduced copurification of SNHG lncRNAs with ZFC3H1 in PABPN1-deficient cells [58]. Also consistent with PABPN1-dependent loading of PAXT onto target transcripts is our PDB-MS data indicating that the proximity between ZC3H14 and PAXT is reduced in the absence of PABPN1 (Fig. 2). In contrast, PABPN1-dependent loading of ALYREF at the 3′ end of mRNAs [59] may sanction transcript for export into the cytoplasm. Although the underlying specificity of PABPN1-dependent nuclear retention remains to be elucidated, we speculate that hyperadenylation mediated by PABPN1 [14] is a key element impeding nuclear export kinetics that would favor ZFC3H1-MTR4 recruitment onto PAXT substrates. ZFC3H1 may further promote RNA hyperadenylation via stimulation of PAP activity, as recently demonstrated for the homologous MTREC complex in fission yeast [60] as well as the copurification of human PAP with PAXT components [61]. RNA hyperadenylation coupled with PAXT recruitment to such PABPN1-bound nuclear-retained transcripts may act as a checkpoint for 3′-5′ exonucleolytic degradation by the RNA exosome. In the absence of PABPN1 and hyperadenylation, polyadenylated PAXT substrates may be bound by competing nuclear PABPs such as ZC3H14 and/or shuttling cytoplasmic PABPs such as PABPC1 [62], and favor nuclear export.

Although we have not definitively proven that PABPN1 and ZC3H14 share binding to poly(A) tails of target transcripts, our protein interaction analyses (Fig. 1) and functional data (Figs 2–6) support this model. Interestingly, the antagonistic roles of ZC3H14 and PABPN1 in nuclear RNA decay is reminiscent of the situation in the fission yeast *S. pombe*, where homologs Nab2 and Pab2, respectively, were shown to have opposing roles in gene regulation [23]. Specifically, *S. pombe* Nab2 protects the inefficiently spliced *rpl30-2* pre-mRNA from Pab2/exosome-mediated nuclear decay alike the set of lncRNAs that are reported here, which are protected from PAXT-dependent decay by ZC3H14. It therefore appears that the antagonistic roles of PABPN1 and ZC3H14 in the control of exosome-mediated nuclear RNA surveillance is evolutionarily conserved. Corroborating this idea is the fact that knockdowns of PABPN1 and ZC3H14 have opposite effects on poly(A) tail length control [32]. How ZC3H14 impedes the function of PABPN1 in PAXT-dependent RNA decay remains to be determined, however. The significant increase in the nuclear-to-cytoplasmic ratio of *SNHG19* lncRNA in the double PABPN1/ZC3H14 depletion compared to the single PABPN1 knockdown (Fig. 6) suggests a mechanism linked to RNA transport, whereby ZC3H14 licences the spliced *SNHG19* lncRNA for nuclear export. Consistent with this idea is previous work linking human ZC3H14 and its budding yeast homolog, Nab2, in the quality control of RNA splicing and export [30,33,51]. Indeed, as lncRNAs expressed from SNHGs are capped, spliced, and polyadenylated like any other mRNA, it will be interesting to consider what features of SNHG lncRNAs prompt PABPN1-dependent nuclear retention and regulation by ZC3H14.

Why is it important to sort nuclear transcripts between productive export and retention-dependent nuclear decay? Pervasive transcription by RNA polymerase II results in the production of a large assortment of nuclear transcripts that are not destined for translation in the cytoplasm, including many lncRNAs. Export of these lncRNAs to the cytoplasm could potentially hijack the translation machinery and perturb protein synthesis by inhibiting mRNA translation and producing aberrant toxic proteins. Although some mechanisms of lncRNA nuclear retention have been described [63], how lncRNAs are retained in the nucleus of eukaryotic cells remains poorly understood. Our study disclosed PABPN1 as an additional factor essential for the nuclear retention of unstable polyadenylated transcripts destined for exosome-mediated RNA decay via PAXT. Additionally, our findings provide strong evidence that the functional interplay between human nuclear PABPs is fundamental to posttranscriptional gene regulation. In this regard, it may be valuable to consider that human disorders associated with mutations in *ZC3H14*, which cause a form of intellectual disability [24], and in *PABPN1*, with give rise to oculopharyngeal muscular dystrophy [64], may be linked to the disruption of this dynamic interplay between nuclear PABPs.

## Supplementary Material

gkaf060_Supplemental_Files

## Data Availability

All the proteomic data have been deposited to the PRIDE ProteomeXchange consortium under the accession PXD0052148. The raw and processed sequencing data (RNA-seq) generated for this study have been submitted to the NCBI Sequence Read Archive (SRA) under the accession number PRJNA1108243.
