# Supplementary material for: Antagonistic roles by the conserved nuclear poly(A)-binding proteins PABPN1 and ZC3H14 in nuclear RNA surveillance
Source: Nucleic Acids Res. 2025 Feb 3;53(3):gkaf060. doi: 10.1093/nar/gkaf060 (PMC11788927; doi:10.1093/nar/gkaf060)
Supplement: gkaf060_Supplemental_Files [file gkaf060_supplemental_files.zip › Supplementary Information.pdf]

The conserved nuclear poly(A)-binding proteins ZC3H14 and PABPN1 have antagonistic roles in nuclear RNA surveillance

Mélodie Latour, Lauren Kwiatek, Anne-Marie Landry-Voyer, and François Bachand[#].

[1]RNA Group, Dept of Biochemistry & Functional Genomics, Université de Sherbrooke, Sherbrooke, Québec, Canada.

SUPPLEMENTARY INFORMATION INVENTORY

- *Supplementary Tables :*
  - Supplementary Table 1
  - Supplementary Table 2
  - Supplementary Tables 3-10 are Excel spreadsheet documents

- *Supplementary Figures :*
  - Supplementary Figures 1-11

Supplementary Table 1. List of small interfering RNAs (siRNA) used in this study.

siRNA	Sequences (5' – 3')
**siGENOME Non-Targeting siRNA#4 (siControl) Dharmacon**	D-001210-04-05
**PABPN1#5**	GGAACGGCCUGGAGUCUGAUU
**ZC3H14**	UGUUUGUUUGUUCACCCAAAUUGUAdTdT
**RBM26**	GUACCUACUUUGAGCAGCA[dT][dT]
**RBM27**	CUGAUCAACUUGAUGUCUU[dT][dT]
**UAP56**	CCUGAUGAGAUAGACAUCUCCUCCUdTdT
**NXF1**	L-013680-01-0005 (Horizon Discovery)

Supplementary Table 2. List of oligonucleotides used for RT-qPCR in this study.

Target	Forward (5' – 3')	Reverse (5' – 3')
GAPDH	GTCAGCCGCATCTTCTTTTG	GCGCCCAATACGACCAAATC
Myc	GATTCTCTGCTCTCCTCGAC	TTCTTGTTCCTCCTCAGAGTC
NALT1	GAGGGAAGCTGAGGCTCAG	CCTACTGGATGACAGCAGC
RPS2	TATGCCAGTGCAGAAGCAGACC	CCTCCTTGGAGCACTTAACAC
SNHG19	ACGATCTTGGGACGAACTGA	AGGGACAAAGTGGTGCGTAG
***SNHG19* spliced**	GACGAACTGAGCCACGAGCG	GCGACGAAACCTGCAGGTAG
***SNHG19* unspliced**	GGAGCTGAGAGTGGAAACAGG	CCCTCCGGATTGAGGTTTACT
RBM27	AAGACAAAAACGGAGGCCCA	CTGAGGACAGCCTCTTGTGG
LINC01273	GAAGAACATTCCAACACAGAC	CTCATACAACATTGTCCTTCTG
**18S ribosomal RNA**	AAACGGCTACCACATCCAAG	CCTCCAATGGATCCTCGTTA

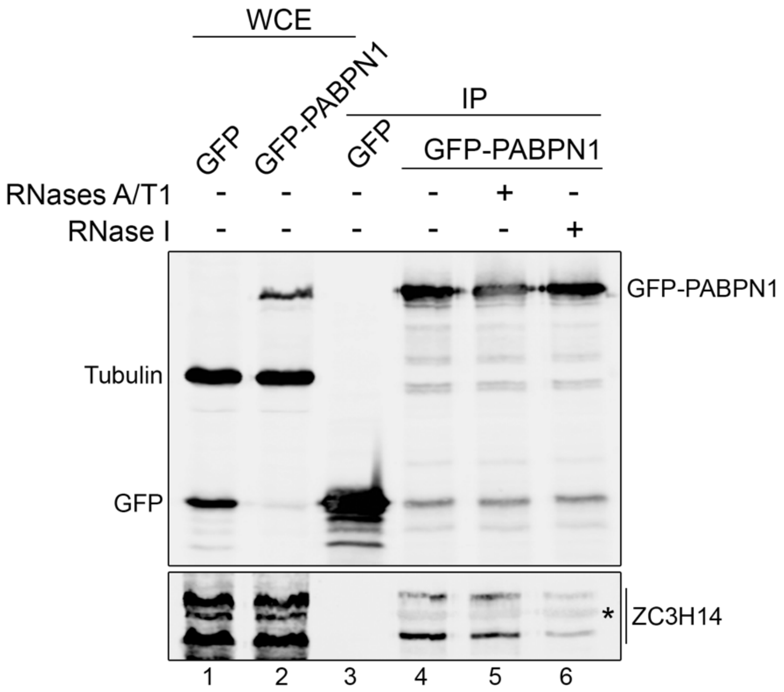

Supplementary Figure 1. Copurification of endogenous ZC3H14 with GFP-PABPN1 is sensitive to RNase I, but not to RNases A/T1. Western blot analysis of total extracts (lanes 1-2) and GFP immunoprecipitates (lanes 3-6) prepared from stable HEK293T cells expressing GFP (lanes 1 and 3) and GFP-PABPN1 (lanes 2 and 4-6). GFP-PABPN1 precipitates were either treated (lanes 5-6) or not treated (lane 4) with the indicated RNases to digest cellular RNAs on beads. The asterisk indicates a non-specific protein that cross-reacts with the ZC3H14 antibody.

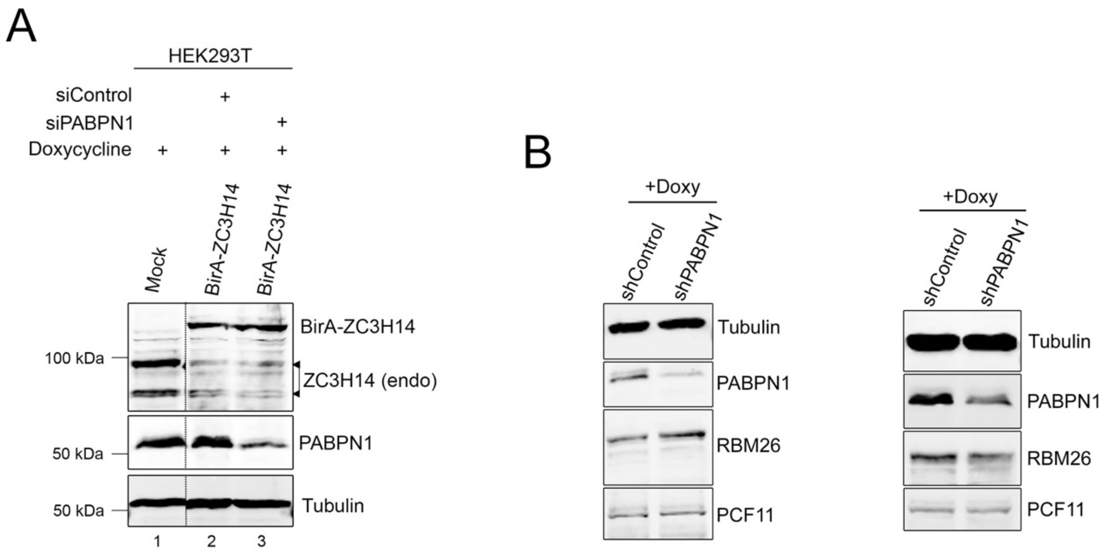

Supplementary Figure 2. siRNA-mediated depletion of PABPN1 in HEK293T cells expressing BirA-tagged ZC3H14. (A) Western blot analysis of total extracts prepared from HEK293T cells that conditionally induce BirA-ZC3H14 (after doxycycline addition) and that were previously transfected with control non-target (lane 2) and PABPN1-specific (lane 3) siRNAs. Control cells were not transfected (lane 1). The antibodies used for Western blot analysis are shown on the right and molecular weights markers on the left. The data in lanes 1 to 3 were from the same blot, with the dashed line indicating some intervening lanes that were cropped out. (B) Western blot analysis of total cell extracts prepared from HeLa cells that conditionally express (+Doxycycline) a nontarget control shRNA (lane 1) or a PABPN1-specific shRNA (lanes 2). The antibodies used for Western blot analysis are shown on the right. Representative blots from two independent depletion experiments are shown.

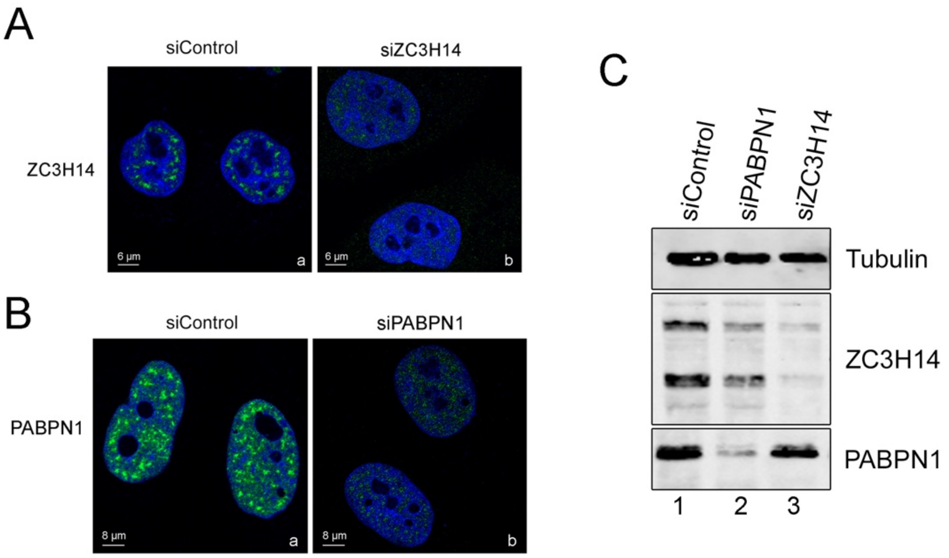

Supplementary Figure 3. Validation of antibody specificity for immunofluorescence analysis of endogenous ZC3H14 and PABPN1. (A) Immunostaining analysis of fixed and permeabilized HeLa cells that were previously transfected with control (a) and ZC3H14-specific (b) siRNAs were stained with antibodies specific for ZC3H14 (a and b). DNA was stained with DAPI to show the nucleus (a and b). Bar, 6 μm. (B) Immunostaining analysis of fixed and permeabilized HeLa cells that were previously transfected with control (a) and PABPN1-specific (b) siRNAs were stained with antibodies specific for PABPN1 (a and b). DNA was stained with DAPI to show the nucleus (a and b). Bar, 8 μm. (C) Western blot analysis of total cell extracts prepared from cells previously transfected with siRNAs specific to PABPN1 (lane 2), ZC3H14 (lane 3), or a nontarget control siRNA (lane 1). The antibodies used for Western blot analysis are shown on the right.

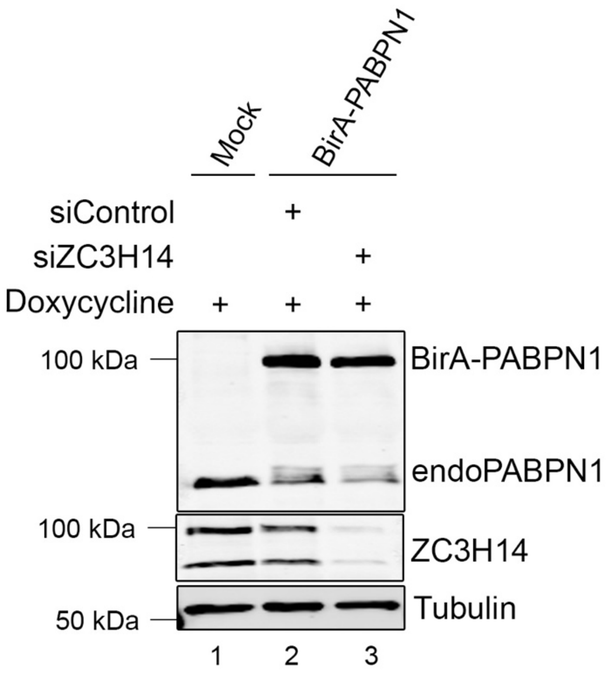

Supplementary Figure 4. siRNA-mediated depletion of ZC3H14 in HEK293T cells expressing BirA-tagged PABPN1. Western blot analysis of total extracts prepared from HEK293T cells that conditionally induce BirA-PABPN1 (lane 2-3) and control cells (lane 1) that were previously transfected with control non-target (lane 2) and ZC3H14-specific (lane 3) siRNAs. Control cells were not transfected (lane 1). The antibodies used for Western blot analysis are shown on the right and molecular weights markers on the left.

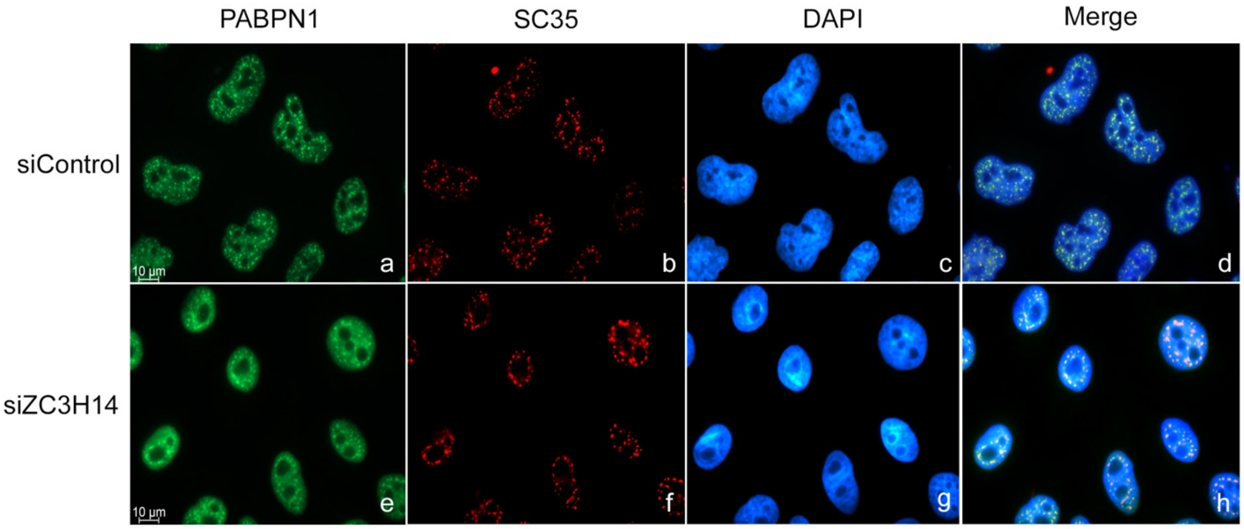

Supplementary Figure 5. **ZC3H14 deficiency reduces PABPN1 signal in nuclear speckles**. Immunostaining analysis of fixed and permeabilized HeLa cells that were previously transfected with control (a-d) and ZC3H14-specific (e-h) siRNAs were stained with antibodies specific for PABPN1 (a and e) and SC35 (b and f). DNA was stained with DAPI to show the nucleus (c and g). Images a and b, and e and f were merged to form d and h, respectively. Bar, 10 μm.

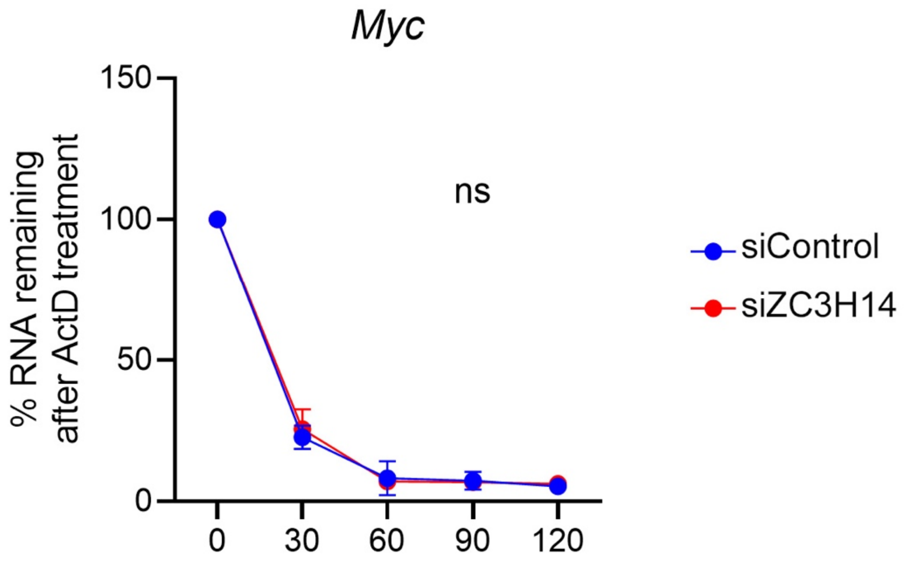

Supplementary Figure 6. ZC3H14 depletion does not affect the stability of the *Myc* mRNA. HeLa cells previously transfected with control (blue circles; siNT) and ZC3H14-specific (red squares; siZC3H14) siRNAs were treated with 5 mg/ml actinomycin D, and RNA was isolated at the indicated time points. The degradation rate of the *Myc* mRNA was determined by RT-qPCR analysis and normalized to *18S rRNA*. Data and error bars represent the mean and standard deviation of three independent experiments. ns, not significant.

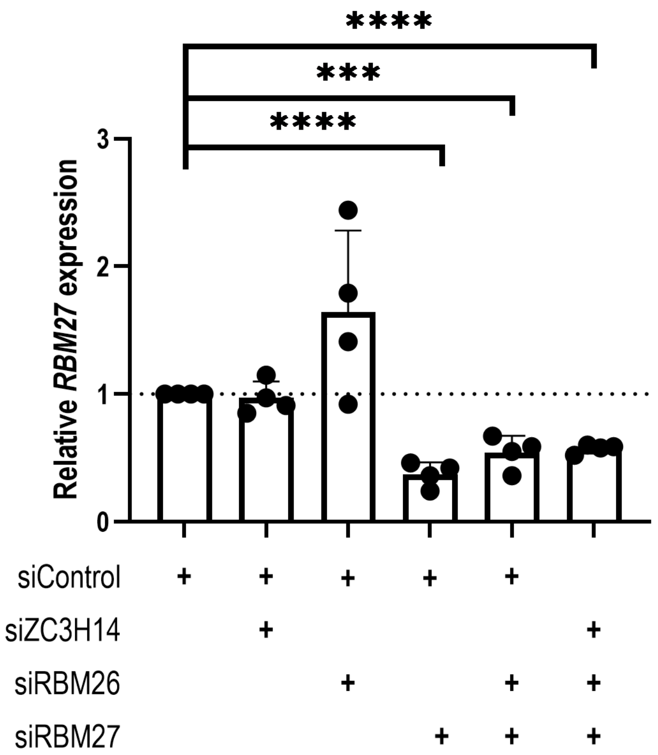

Supplementary Figure 7. RT-qPCR analysis of *RBM27* mRNA levels. RT-qPCR analysis of *RBM27* mRNA from total RNA harvested from HeLa cells that were previously transfected with the indicated siRNA combinations. Data and error bars represent the means and standard deviations of four independent experiments, respectively. P-values (∗∗∗) ≤0.001; (∗∗∗∗) ≤0.0001 were determined with an unpaired Student t test.

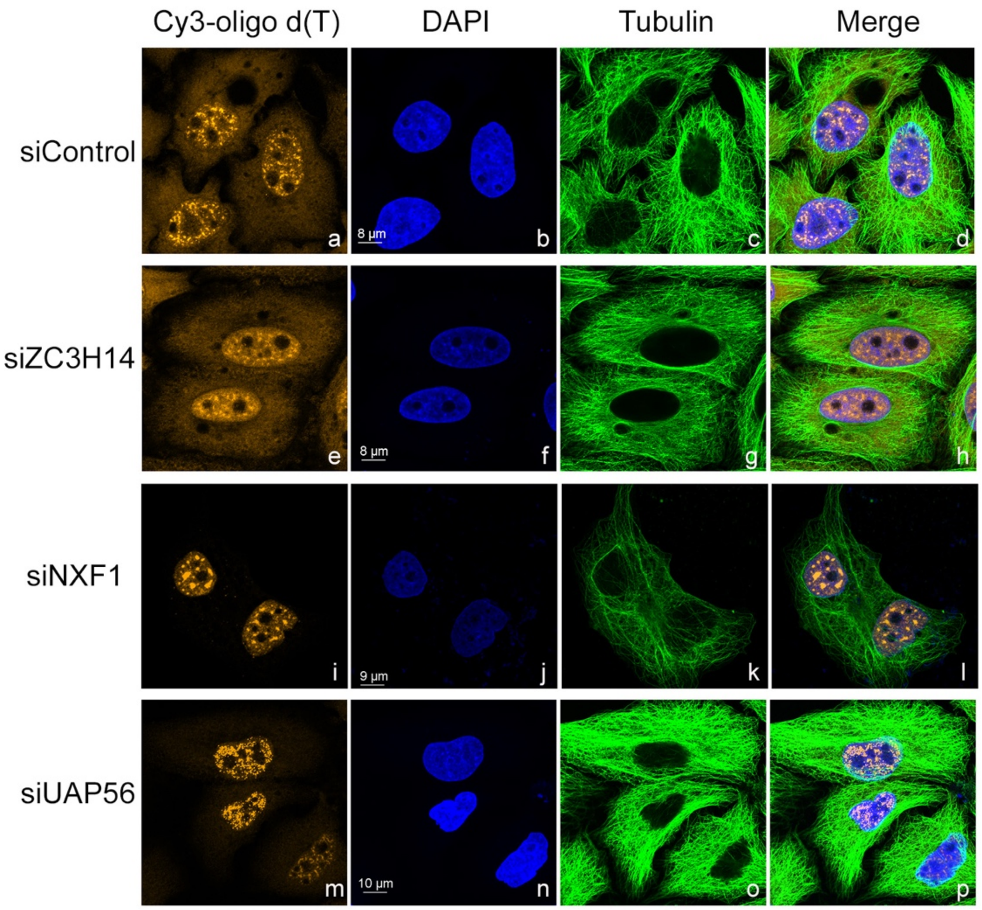

Supplementary Figure 8. ZC3H14 is not a general RNA export factor. Deconvoluted images of HeLa cells that were previously transfected with control (a–d), ZC3H14-specific (e–h), NXF1-specific (i–l), and UAP56-specific (m–p) siRNAs were simultaneously analyzed by FISH using a Cy3-labeled oligo d(T) probe for polyadenylated RNAs (a, e, i, m) and immunostaining for the cytosolic tubulin (c, g, k, o). DNA stained with DAPI shows the nucleus of each cell (b, f, j, n). Scale bar sizes are indicated.

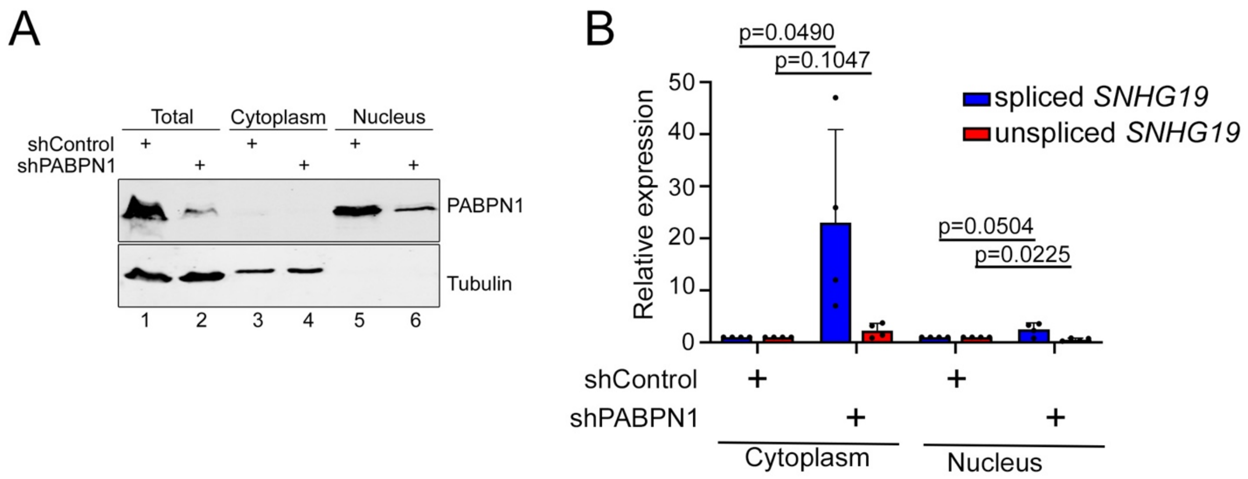

Supplementary Figure 9. Cytoplasmic accumulation of the spliced *SNHG19* lncRNA in PABPN1-deficient cells. (A) Western blot analysis of total extracts (lanes 1-2) as well as of cytoplasmic (lanes 3-4) and nuclear (lanes 5-6) fractions prepared from cells induced (using Doxycycline) to express PABPN1-specific shRNAs (lanes 2, 4, and 6) or a control nontarget shRNA (lanes 1, 3, and 5). (B) RT-qPCR analysis of spliced (blue) and unspliced (red) versions of the *SNHG19* lncRNA using RNA prepared from cytoplasmic and nuclear fractions of cells previously induced to express PABPN1-specific shRNAs (shPABPN1) or a control nontarget shRNA (siControl). Data and error bars represent the means and standard deviations of four independent experiments, respectively. P-values are indicated and were determined with an unpaired Student t test.

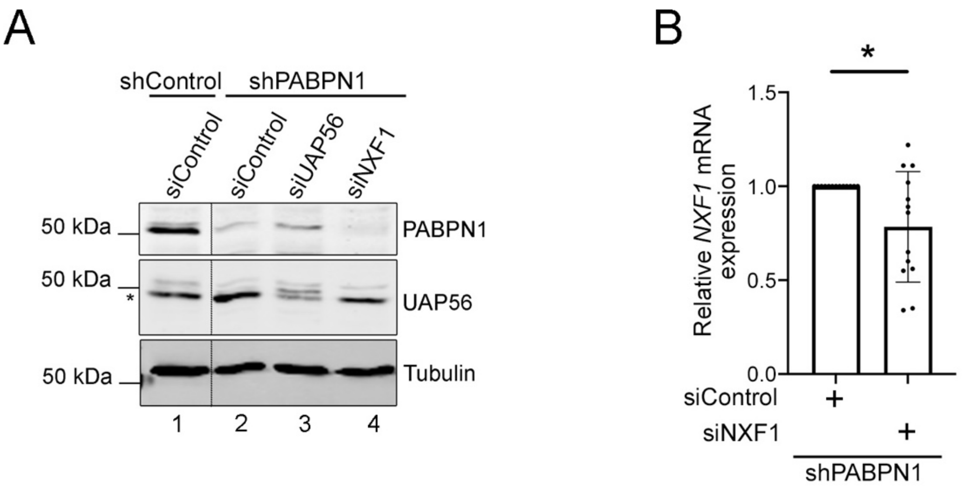

Supplementary Figure 10. Depletion of UAP56 and NXF1 in PABPN1-deficient cells. (A) Western blot analysis of total cell extracts prepared from HeLa cells that conditionally express (+Doxycycline) a nontarget control shRNA (lane 1) or a PABPN1-specific shRNA (lanes 2-4) and that were previously transfected with siRNAs specific to UAP56 (lane 3), NXF1 (lane 4), or a nontarget control siRNA (lanes 1-2). The antibodies used for Western blot analysis are shown on the right. The data in lanes 1 to 4 were from the same blot, with the dashed line indicating some intervening lanes that were cropped out. (B) RT-qPCR analysis of *NXF1* mRNA from total RNA harvested from HeLa cells that conditionally express (+Doxycycline) a PABPN1-specific shRNA and that were previously transfected with control nontarget (siControl) or NXF1-specific siRNAs. Data and error bars represent the means and standard deviations of independent experiments, respectively. P-value (∗) ≤0.05 was determined with an unpaired Student t-test.

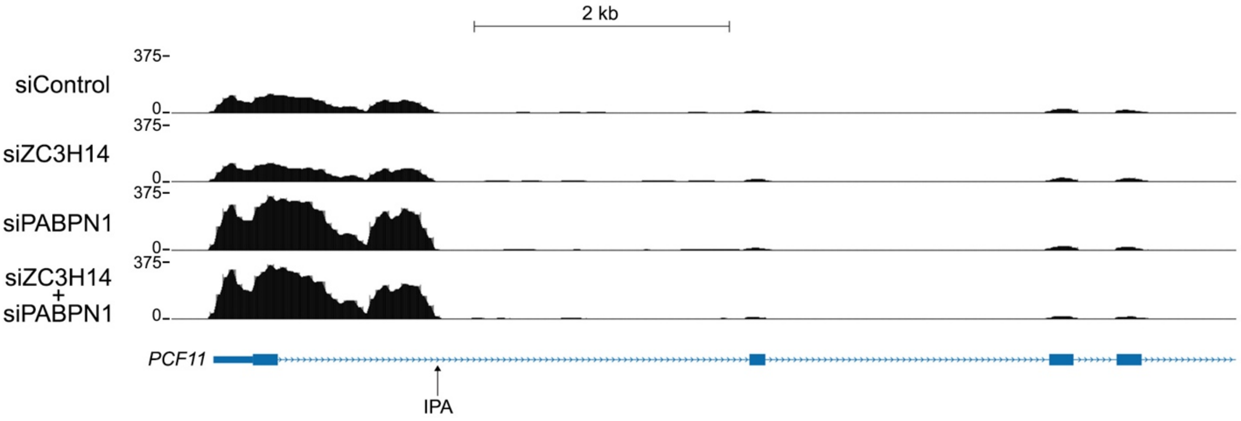

Supplementary Figure 11. *PCF11* ptRNA accumulates in PABPN1-deficient cells, but is not affected by ZC3H14 deficiency. Read coverage over the *PCF11* genes from RNA-seq data of HeLa cells previously transfected with the indicated siRNAs. Transcript annotations are from GENCODE V44. An arrow indicates the position of the intronic polyadenylation (IPA) sites that results in the expression of an unstable ptRNA that is sensitive to PABPN1-dependent nuclear decay.
